# Steam-Assisted Ammonolysis of MoO_2_ as a Synthetic Pathway to Oxygenated δ-MoN

**DOI:** 10.3390/ma18102340

**Published:** 2025-05-17

**Authors:** Shobhit Pandey, Elise A. Goldfine, Shriya Sinha, Chi Zhang, Jill K. Wenderott, Lucien Kaczmarczyk, Ksawery Dabrowiecki, Vinayak P. Dravid, Gabriela B. González, Sossina M. Haile

**Affiliations:** 1 Materials Science and Engineering, Northwestern University, Evanston, IL 60208, USA or shobhitpandey2015@u.northwestern.edu (S.P.); or elisegoldfine@u.northwestern.edu (E.A.G.); or chizhang@u.northwestern.edu (C.Z.); jill.wenderott@drexel.edu (J.K.W.); v-dravid@northwestern.edu (V.P.D.); 2 Physics and Astrophysics, DePaul University, Chicago, IL 60614, USA; shriyasi@umich.edu (S.S.); lucienvkacz@gmail.com (L.K.); kdabrowi@depaul.edu (K.D.)

**Keywords:** reaction pathway, ammonolysis, molybdenum nitride

## Abstract

A common route for the synthesis of molybdenum nitrides is through the temperature-programmed reaction of molybdenum oxides with NH_3_, or ammonolysis. In this work, the role of precursor phase, gas phase chemistry (impact of H_2_O), and temperature profile on the reaction outcome (700 °C) was examined, which resulted in varying amounts of MoO_2_, H_2_MoO_5_, and the nitride phases—cubic γ (nominally Mo_2_N) and hexagonal δ (nominally MoN). The phase fraction of the δ phase increased with precursor in the sequence MoO_2_ > MoO_3_ > H_2_MoO_5_. Steam in the reaction gas also favored the production of δ over γ, but with too much steam, MoO_2_ was obtained in the product. Synthesis conditions for obtaining nearly phase-pure δ were identified: MoO_2_ as the precursor, 2% H_2_O in the gas stream, and a moderate heating rate (3 °C/min). In situ X-ray diffraction provided insights into the reaction pathway. Extensive physico-chemical analysis of the δ phase, including synchrotron X-ray and neutron diffraction, electron microscopy, thermogravimetric analysis, X-ray photoelectron spectroscopy, and prompt gamma activation analysis, revealed its stoichiometry to be MoO_0.108(8)_N_0.892(8)_H_0.012(5)_, indicating non-trivial oxygen incorporation. The presence of N/O ordering and an impurity phase Mo_5_N_6_ were also revealed, detectable only by neutron diffraction. Notably, a computationally predicted MoON phase (doi: 10.1103/PhysRevLett.123.236402), of interest due to its potential to display a metal-insulator transition, did not appear under any reaction condition examined.

## 1. Introduction

Ammonolysis of precursor oxides, that is, temperature-programmed reaction with NH_3_ at high temperature, is a well-established route for the preparation of a broad range of nitrides and especially oxynitrides [1]. Despite the widespread use of this method, challenges arise in ensuring reproducibility because the reaction outcomes, in terms of phase, morphology, and anion chemistry, are highly dependent on the specific reaction conditions such as choice of oxide precursor, temperature profile, gas composition, and gas flow rate. Furthermore, because oxygen and nitrogen are not readily distinguished in X-ray and electron methods, residual oxygen from the ammonolysis of oxide precursors can remain unnoticed. Thus, careful assessment of the reaction product in combination with systematic variation of synthesis parameters is required in order to establish synthetic routes that reproducibly generate target products. The current study centers on the reaction outcomes from the ammonolysis of molybdenum oxide precursors. The preparation of high specific surface area γ-MoO_x_N_y_, a cubic material that is highly desirable for a range of catalytic applications, by ammonolysis of MoO_3_ is well known [2]. In some cases, a competing hexagonal phase, δ-MoN [3], can be found alongside the target product [4,5]. The conditions that favor this phase are not well-established, though it has been noted that the prevalence of δ-MoN depends on the nature of the precursor [4,6]. Additionally, while there has been some attention paid to the presence of oxygen in the cubic γ phase [7,8,9,10,11] and to its impact on properties [12], it is unknown whether oxygen may be a component in δ.

The possibility of oxygen incorporation into molybdenum nitride raises the question of whether anion-ordered molybdenum oxynitride phases, in which oxygen and nitrogen occupy distinct crystallographic sites, can be deliberately prepared. To date, no such compound has been experimentally realized, though we have previously reported preferential site occupancies in γ-MoO_x_N_y_ [7]. This stands in contrast to the behavior in the tantalum system, in which several TaON phases with fully differentiated anion sites (i.e., no mixed occupancies) have been observed experimentally and even more are predicted computationally [13,14]. Ordering of oxygen and nitrogen in the Mo system is particularly intriguing as it has been predicted to generate a material, MoON, that displays a Peierls metal-insulator transition (MIT) [15] in analogy to VO_2_ and NbO_2_ [16]. Above their respective transition temperatures, VO_2_ and NbO_2_ (in which V^4+^ has the 3*d*^1^ electronic configuration and Nb^4+^ the 4*d*^1^ configuration) are metals with conduction bands derived primarily from the partially filled *d* states. Below the MIT, metal–metal dimerization driven by phonon-electron coupling renders the materials insulating. The realization of such a transition in MoON (in which Mo^5+^ has the 4*d*^1^ configuration) would expand the fundamental understanding of metal–insulator behavior and potentially open the door to new applications.

Beyond the hypothetical material MoON, the computational prediction of new compounds has in recent years outpaced synthetic efforts [17,18], motivating experimental studies that clarify the effect of synthetic parameters on the nature of the product phases. In the case of nitrides, impressive progress has been made in realizing computationally predicted cation-ordered ternary compounds [19,20]. Achieving control over the extent of oxygen incorporation, detecting the oxygen levels, and ensuring anion ordering in oxynitrides are all substantially more difficult than encountered in the preparation of cation-ordered ternary nitrides. Not only are nitrogen and oxygen chemically similar, the anions are volatile at typical synthesis conditions. Even in those oxynitrides in which the stoichiometry is well-controlled such as Zr_4_O_5_N_2_ [21], Zr_2_ON_2_ [22,23], SrTaO_2_N [24], and BaTaO_2_N [24], the anions are not fully ordered, if at all, and many computationally predicted anion-ordered oxynitrides with potentially transformative properties [18] remain unrealized.

For the synthesis of MoON, one can anticipate, by analogy to the Ta system and the well-established route for producing TaON rather than Ta_3_N_5_ [14,25], that the introduction of steam along with the supplied ammonia will generate the desired product. In principle, steam provides control over the oxygen chemical potential and accordingly control over the O:N ratio on the product. To date, few studies have examined the role of steam in the ammonolysis of molybdenum oxide precursors. In one case, ammonolysis of MoO_3_ in the presence of steam has been utilized to influence the morphology of the product, but surprisingly little effect has been observed on the specific phase obtained [6].

In the present work, we examine the outcomes resulting from the reaction of three different precursors—MoO_3_, MoO_2_, and H_2_MoO_5_—and from the presence of H_2_O in the reactant ammonia gas. While the hypothetical MoON phase was not detected, it was possible to identify conditions that favored the formation of the hexagonal δ phase, a material that has not been previously obtained as the predominant product via the relatively simple ammonolysis approach. With the synthesis route established, we perform extensive characterization of δ-MoO_x_N_y_ to establish its complete chemical and structural nature. In particular, we quantify the residual oxygen content, the extent of anion ordering, and the extent of hydrogen incorporation, factors that would be expected to influence the properties of the material. Due to the significant oxygen content, it is referred to as δ-MoO_x_N_y_ or, when explicitly considering the small but non-zero hydrogen content, as δ-MoO_x_N_y_H_z_. The chemistry is thus similar to that of γ-MoO_x_N_y_ [7], in which residual oxygen and incorporated hydrogen produce a material with stoichiometry, which differs from the conventionally identified chemistry of Mo_2_N. In further similarity to γ-MoO_x_N_y_, the anions in δ-MoO_x_N_y_ are partially, but not fully, ordered.

## 2. Survey of Ammonolysis Outcomes

Nine types of ammonolysis conditions were employed, using a combination of the three different precursors noted above (α-MoO_3_, MoO_2_, and H_2_MoO_5_), four different gas atmospheres (reflecting different levels of humidification), and two different heating profiles, Table 1. The reagents MoO_3_ (99.9995% metal basis) and MoO_2_ (99% metal basis) were purchased from Alfa Aesar. The peroxo molybdate H_2_MoO_5_ (more formally designated as MoO_2_(O_2_)H_2_O) was synthesized in-house by oxidation of MoO_3_ using the aqueous H_2_O_2_ solution as described earlier [2,26,27]. The phases of the precursors were confirmed by X-ray powder diffraction (SI Figure S1). Ammonolysis was carried out in a horizontal tube furnace using 0.125 g of material and an ammonia flow rate of 100 sccm (standard cubic centimeters per minute). Steam was supplied in selected experiments by supplying Ar saturated with H_2_O at 25 °C (0.031 atm *p*H_2_O) along with the ammonia. By selecting various flow rates of the humidified Ar (with fixed NH_3_ flow rate), the steam concentration was varied between values of 1.5 and 2.5%. The reactant gas was introduced into the sample environment at room temperature and the sample was then subjected to the desired temperature program. In all but one case, the following default heating profile was utilized: the sample was heated to 700 °C at 3 °C/min, held for 12 h, and then cooled (over approximately 6 h at the furnace natural cooling) to ambient temperature while still under ammonia flow. In the remaining case, the impact of including a slow ramp at intermediate temperatures (0.6 °C/min between 350 °C and 600 °C) on the ammonolysis of MoO_3_ with a gas stream of 2% H_2_O was examined. Heating profiles of this type are often utilized in dry ammonolysis of MoO_3_ because they tend to yield phase-pure γ-MoO_x_N_y_ of high specific surface area [2,7,27]. In all cases, after reaching room temperature, the chamber was purged with N_2_ for 30 min before exposing the sample to air. The products were evaluated ex situ by laboratory X-ray powder diffraction (XRD) analysis and scanning electron microscopy (see Section 5).

**Figure 1:**
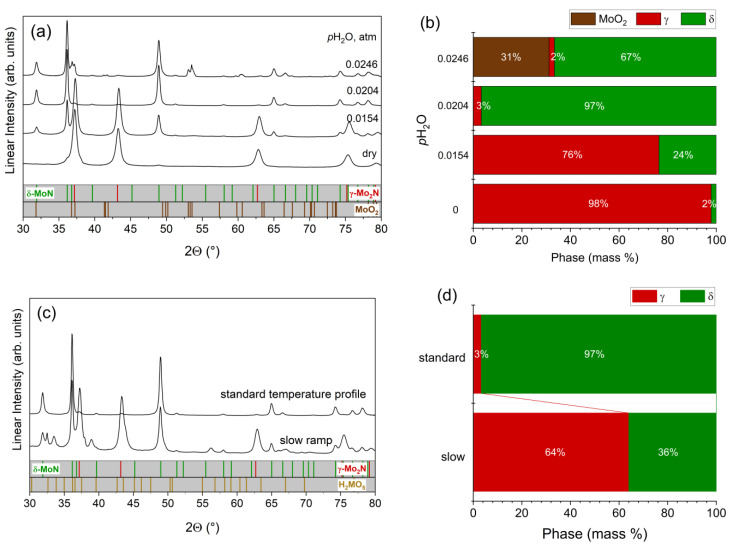
Influence of reaction parameters on ammonolysis outcomes using MoO_3_ as the precursor: (**a**,**b**) influence of steam partial pressure (as indicated) using the standard heating protocol; (**c**,**d**) influence of heating protocol (standard, and slow ramp, as indicated) using *p*H_2_O = 0.0204 atm. Laboratory X-ray diffraction patterns are shown in (**a**,**c**) and refined phase fractions are shown in (**b**,**d**). Data are collected using Cu Kα radiation (λ = 1.5406 Å).

Illustrated in Figure 1 is the remarkable influence of steam on the reaction outcome of the ammonolysis of MoO_3_. Dry ammonolysis using the default heating profile resulted in the formation of nearly, but not entirely, phase-pure γ-MoO_x_N_y_. Detection of a small amount of δ-MoO_x_N_y_ under these conditions is consistent with literature reports that slow ramp rates are required to achieve phase-pure γ-MoO_x_N_y_ from the ammonolysis of MoO_3_. With increasing *p*H_2_O from 0 to 0.020 atm (Figure 1a,b), the mass percentage of δ-MoO_x_N_y_ in the product mixture increased dramatically, from about 2% to 97%. A further increase in *p*H_2_O to 0.025 atm favored the formation of MoO_2_, largely at the expense of δ. The appearance of the dioxide presumably reflects the increase in oxygen chemical potential with an increase in *p*H_2_O. The use of a slow heating rate (with *p*H_2_O = 0.02) suppressed the formation of δ-MoO_x_N_y_ and enhanced the formation of γ-MoO_x_N_y_ (Figure 1c,d), a result that mirrors the behavior of MoO_3_ when ammonolysis is performed under dry conditions. The observation here that intermediate concentrations of steam and high heating rates favor the formation of δ-MoO_x_N_y_ may be interrelated as the heating rate will impact the rate at which steam is generated as a reaction byproduct. This mirrors the observation that low heating rates and high gas flow rates in the preparation of the cubic γ phase are advantageous for obtaining high surface area products because the generated H_2_O can otherwise induce sintering [5].

**Figure 2:**
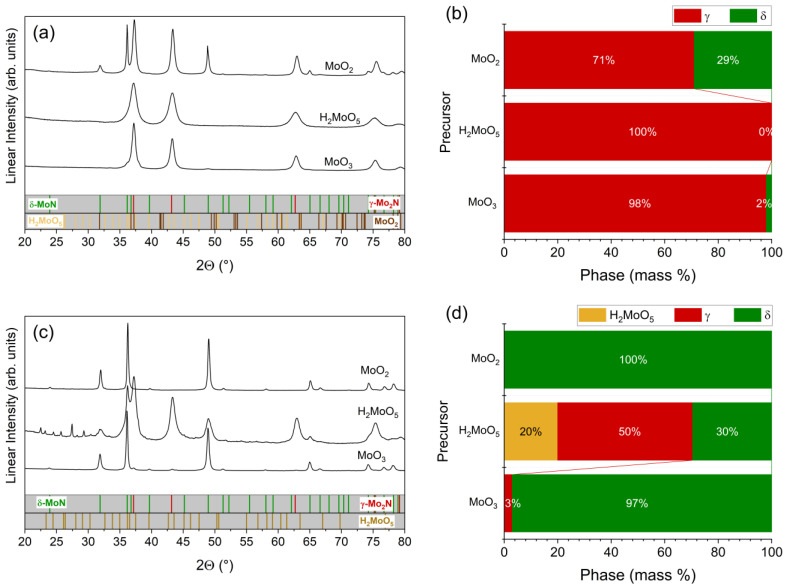
Influence of precursor and H_2_O on ammonolysis outcomes: (**a**,**b**) influence of precursor (as indicated) under anhydrous conditions; (**c**,**d**) influence of precursor (as indicated) under hydrous conditions using a steam partial pressure of 0.0204 atm (2.04% steam). Laboratory diffraction patterns are shown in (**a**,**c**) and refined phase fractions are shown in (**b**,**d**). Data are collected using Cu Kα radiation (λ = 1.5406 Å).

The choice of the precursor also strongly influenced the nature of the resulting phases, Figure 2, with the phase fraction of δ-MoO_x_N_y_ increasing in the sequence MoO_2_ > MoO_3_ > H_2_MoO_5_. Under conventional anhydrous conditions, the precursor H_2_MoO_5_ produced only γ-MoO_x_N_y_, consistent with prior studies of this reagent [27,28], whereas MoO_3_ and MoO_2_ reagents yielded, respectively, 2 and 29 mass % of δ-MoO_x_N_y_ in the product phases. The production of a mixture of δ-MoO_x_N_y_ and γ-MoO_x_N_y_ from dry ammonolysis of MoO_2_ agrees with our prior in situ study of this precursor, where it was found that the two product phases co-existed between ~675 and 800 °C, with the precursor being completely consumed at ~700 °C [29]. Jaggers et al. similarly observed that ammonolysis of MoO_2_ resulted in a mixture of γ and δ phases under conditions that produced only γ from MoO_3_ [4]. Introduction of 2% steam increased the phase fraction of δ-MoO_x_N_y_ regardless of the precursor (as already noted for MoO_3_). In the case of H_2_MoO_5_, the product mixture following hydrous ammonolysis contained 30 mass % δ-MoO_x_N_y_, along with 50 mass % γ-MoO_x_N_y_ and unreacted precursor. Thus, steam both suppressed ammonolysis of H_2_MoO_5_ and shifted the nature of the product from γ-MoO_x_N_y_ towards δ-MoO_x_N_y_. Hydrous ammonolysis of MoO_2_, resulted in phase-pure δ-MoO_x_N_y_ (at the level of impurity phase detection by laboratory XRD), a dramatic shift from only 29 mass % of this phase under anhydrous conditions. While higher steam concentrations were not examined for these precursors, because ammonolysis of MoO_3_ under higher steam concentrations (2.46%) yielded MoO_2_ as a major product, it can be readily concluded that higher steam when using MoO_2_ as the reagent would also result in the presence of the dioxide in the product.

**Figure 3:**
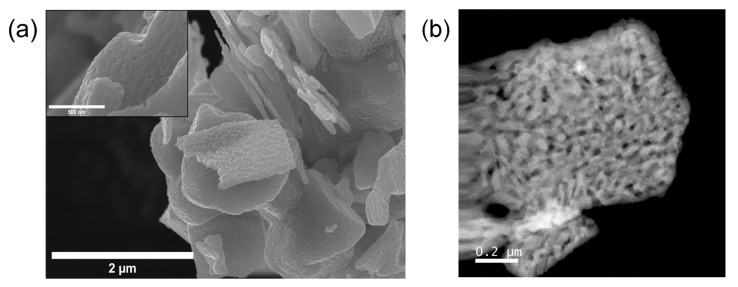
Electron microscopy images of representative δ-MoO_x_N_y_ particles produced by ammonolysis of MoO_2_ in the presence of steam (Table 1, condition 7): (**a**) scanning electron microscopy image, and (**b**) transmission electron microscopy image.

In sum, the conditions that favor the formation of δ-MoO_x_N_y_ are the use of MoO_2_ as a precursor, a relatively high heating rate, and an intermediate concentration of steam in the gas (condition 7 in Table 1); too low a steam content leads to γ-MoO_x_N_y_ and too high presumably retains MoO_2_. The diffraction pattern shown in Figure 2c corresponding to these reaction conditions, termed steam-assisted ammonolysis of MoO_2_, is matched to the *P6_3_mc* structure reported by Bull et al. [3] for δ-MoN, with lattice constants *a* ≈ 5.730 Å and *c* ≈ 5.608 Å. Ganin et al. [30] have identified this phase as the δ_3_ variant amongst several hexagonal MoN phases. The reaction converts the dark brown MoO_2_ precursor into a shiny black product and creates a dimpled texture on the resulting particles, while leaving their macroscopic shape unperturbed, Figure 3a. It has been suggested that steam induces volatilization during nitridation of MoO_3_ [6] and a similar phenomenon may be responsible for the texturing observed here. The crystallite size as determined from the peak broadening in the diffraction pattern is ~33 nm, which is too small to be observed in the SEM (scanning electron microscopy) image, but can be recognized in the TEM (transmission electron microscopy) image, Figure 3b.

With the synthesis route for obtaining δ-MoO_x_N_y_ established, further investigations of this material were carried forward in two ways. (1) The influence of steam on the transformation of MoO_2_ under reaction with ammonia was further studied by using in situ X-ray diffraction analysis. (2) The physico-chemical properties of δ-MoO_x_N_y_ (following synthesis in a conventional tube furnace by condition 7) were characterized. It is to be noted that while laboratory XRD analysis demonstrated a high level of phase purity, subsequent synchrotron and neutron studies, detailed below, indicated the presence of small concentrations of additional molybdenum nitride phases. As characterized by laboratory XRD, the reaction was quite reproducible, with the impurity γ-MoO_x_N_y_ (<2%) occasionally observed in the product (Supplementary Materials Figure S2). Samples with detectable amounts of the undesired phase were not utilized for further characterization.

## 3. In Situ Transformation of MoO_2_ to Nitrogen-Bearing Phases

In situ diffraction experiments for studying the reaction pathway during MoO_2_ ammonolysis were performed using an Anton Paar XRK 900 reactor chamber (Graz, Austria) mounted onto a 9 kW Cu rotating anode Rigaku Smartlab XE diffractometer (Osaka, Japan) and equipped with a D/TEX Ultra 250 1D silicon strip detector (Rigaku, Osaka, Japan). Measurements were taken in θ/2θ geometry over the angular range 11–82° with a scan speed of 50°/min to minimize dwell time at each temperature. MoO_2_ was placed inside the reactor chamber at ambient temperature, purged with N_2_, and subjected to a flow of NH_3_ and He bubbled through a room temperature water bath to achieve 2% *p*H_2_O in the ammonolysis stream. The chamber is configured such that the gas flows upwards through the sample, which is in turn placed as a thin layer of powder atop a porous support. In this way, thermal and compositional gradients during the reaction are minimized. The material was subjected to a thermal profile that mimicked the ex situ studies: heat to 700 °C (3 °C/min, 10–12 h hold) and cool to ambient temperature (10 °C/min) while still under gas flow. At the completion of the experiment, the chamber was purged with N_2_ for 30 min before exposing the sample to air.

**Figure 4:**
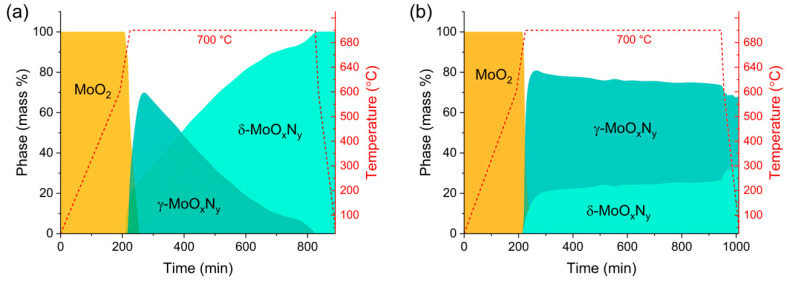
Phase evolution upon the ammonolysis of MoO_2_ as determined from the analysis of XRD patterns collected in situ: (**a**) under hydrous conditions, using 2.04% steam, and (**b**) under anhydrous conditions.

The results of the reaction pathway studies, Figure 4 (see also Supplementary Materials Figure S3), revealed that under both dry and humidified ammonia, the product phases δ-MoO_x_N_y_ and γ-MoO_x_N_y_ appeared simultaneously, with these phases first detected at a slightly lower temperature in the humidified environment (670 °C) than the dry environment (690 °C). In both experiments, consumption of the precursor occurred rather rapidly, with MoO_2_ being completely absent within a few minutes of reaching 700 °C. The precursor is just barely detected upon reaching the hold temperature and is absent in the next measurement, recorded after 30 min. Further similarity between the two conditions is evident in the initial phase assemblage, with the cubic γ-MoO_x_N_y_ phase being the dominant product at the initial stages of reaction. Differences between the two results emerge during the 700 °C hold. Over this period, the phase fraction of γ-MoO_x_N_y_ in the humidified experiment peaks then gradually declines until only δ-MoO_x_N_y_ remains. In contrast, under dry conditions, the phase fraction of cubic γ-MoO_x_N_y_ only gradually decreases towards a plateau value (of approximately 75 mass %) after attaining a peak value of ~82 mass %. Thus, the presence of H_2_O in the gas stream does not appear to facilitate the direct transformation from MoO_2_ to δ-MoO_x_N_y_ in a manner that bypasses γ-MoO_x_N_y_. Instead, it appears to catalyze the transformation from γ-MoO_x_N_y_ to δ-MoO_x_N_y_.

The results for the dry condition are in agreement with our earlier study in which the reaction pathway was examined using a hold temperature of 800 °C [29]. In that case, the two competing product phases were also observed at 690 °C. The higher temperature exposure, however, resulted in the formation of phase-pure γ-MoO_x_N_y_ in a reaction that was largely complete by the time the system reached the temperature of 800 °C. Thus, higher reaction temperatures favor γ-MoO_x_N_y_ formation over δ-MoO_x_N_y_ when the precursor is microcrystalline MoO_2_.

## 4. Physico-Chemical Characterization of δ-MoO_x_N_y_

The physico-chemical properties of δ-MoO_x_N_y_ were characterized by a range of methods to fully establish the material stoichiometry, morphology, and crystallography. These methods include chemical analysis by a combination of microchemical combustion analysis, neutron prompt gamma activation analysis (PGAA), and thermogravimetric analysis (TGA); microstructure analysis by scanning electron microscopy; BET (Brunauer–Emmett–Teller) surface area measurement; pycnometry for density measurement; X-ray photoemission spectroscopy (XPS) for oxidation state determination and for additional chemical analysis; transmission electron microscopy (TEM) in conjunction with selected area diffraction for additional morphological characterization, along with electron energy loss spectroscopy (EELS) to further refine the chemical analysis and oxidation state analysis. Structure refinement against neutron powder diffraction (NPD) data was performed with the goal of differentiating oxygen and nitrogen, as well as locating any hydrogen. Complete acquisition details are provided in the Characterization Methods section. The results of these comprehensive studies are summarized in Table 2. In the table and hereafter, a number in parentheses following a reported measured value indicates the uncertainty in the final digit(s). The uncertainty is given in the range from 1 to 9, with the exception of crystallographic parameters, for which the uncertainty is reported in the range from 2 to 19.

Considering first the physical characteristics, the macroscopic shape of the produced δ-MoO_x_N_y_ particles, introduced briefly above (Figure 3), was found to reflect the morphology of the precursor MoO_2_; this behavior is also well-documented for γ-MoO_x_N_y_ [29]. However, in comparison to typical γ-MoO_x_N_y_ produced by dry ammonolysis at 700 °C (135–160 m^2^/g [27]), the specific surface area is relatively low, only 30.1(6) m^2^/g, though much higher than the 1 m^2^/g to 2 m^2^/g of the starting precursor MoO_2_. As expected from the difference in specific surface areas, the crystallite size determined from peak broadening is larger in δ-MoO_x_N_y_ than in γ-MoO_x_N_y_ [7], differing by about a factor of five. The relatively high density of δ-MoO_x_N_y_ as measured by pycnometry, 9.01(1) g/cm^3^, suggests the absence of closed porosity, another difference from γ-MoO_x_N_y_, in which low density was interpreted to reflect the presence of internal pores [7].

### 4.1. Chemical Analysis

**Figure 5:**
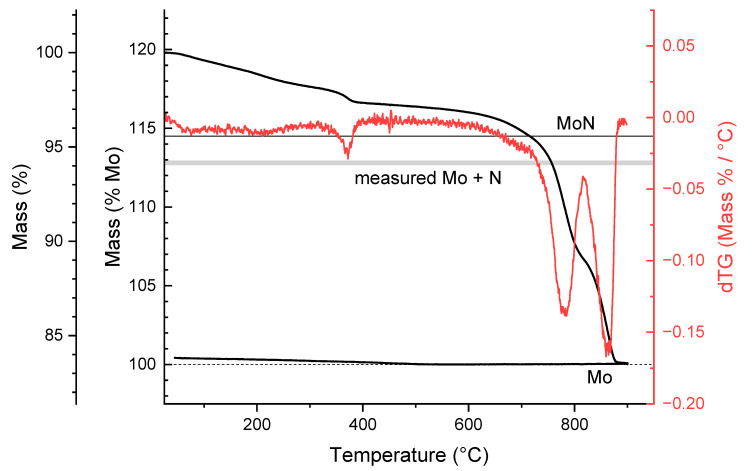
TGA and differential (dTG) mass loss profiles of δ-MoO_x_N_y_ produced by the ammonolysis of MoO_2_ in the presence of steam (Table 1, condition 7) upon exposure to dilute hydrogen (3% H_2_ balance Ar). At the conclusion of the measurement, only Mo remains and the mass % is reported both as % of the initial mass and as % relative to the Mo amount. Horizontal lines indicate the mass of ideal MoN and the mass range implied by the combustion analysis determination of the N content. The difference in mass between ideal MoN and the result deduced from chemical analysis is taken to be due to oxygen. The peak in the dTG profile at ~380 °C is assigned to the loss of surface species. The gradual mass loss from ~400 to 700 °C is assigned to the loss of approximately one monolayer of oxide.

Thermogravimetry under flowing hydrogen (3% H_2_, balance Ar) was used to establish the anion mass in the material, Figure 5. Upon heating to 900 °C (2 °C/min), complete reduction occurred at 875 °C, and no further weight loss was observed during the 3 h hold at 900 °C. The gradual mass loss that occurs between the initiation of the experiment and 400 °C is taken to represent primarily the desorption of surface sorbed species, such as NH_3_, H_2_O, N_2_ and H_2_, that remain following the ammonolysis reaction. This identification is supported by the presence of a small but clear peak in the differential mass profile that completes at ~398 °C. Given the specific surface area of 30.1 m^2^/g, the low-temperature mass loss of ~2.7% corresponds to a surface concentration of one H_2_O or NH_3_ molecule per 3 Å^2^. The reasonableness of this value further supports the identification of the initial mass loss to surface desorption. Additionally, the mass loss under flowing Ar (Figure S4), showed a similar (2.0%) extent of mass loss up to a temperature of 400 °C, as would be expected if only surface desorption occurred under both gases.

From the combustion chemical analysis, the mass fraction of nitrogen in the samples in the as-prepared and surface-desorbed states was directly quantified (Table 2), whereas from the TGA data, the mass % of Mo (83.5 of the total sample and 85.3 following Ar surface desorption) was revealed. Combining these, we find respective Mo:N atomic ratios in the as-prepared and surfaced-desorbed samples of 1.04:1 and 1.13:1. The former matches the PGAA Mo:N ratio of 1.03(3), a method only utilized on the as-prepared material (see Supplementary Materials Figure S5 for the PGAA spectra). The large difference between N content in the as-prepared and surface-desorbed samples suggests that ammonia is the predominant surface species. If true, it implies that the PGAA Mo:H ratio reflects a large quantity of surface H in the form of sorbed NH_3_ molecules. Matching the difference in N content of the as-prepared and surface-desorbed samples entirely to the loss of surface ammonia yields a Mo:H atomic ratio in the as-prepared material of 4.42, slightly larger than the PGAA value of 4.05(3). Thus, the data are consistent with NH_3_ as the predominant surface species, with H_2_O accounting for perhaps 10% of the surface-sorbed molecules. The results also allow for the possibility of bulk hydrogen incorporation, with an upper bound of ~0.02 H per Mo. Though the mass of hydrogen detected by combustion analysis is barely above the uncertainty, and quantitative comparison of the as-synthesized and post-desorption values is unjustified, the results indicate a decrease in this species following surface desorption.

Further evaluation of the material stoichiometry (which provides clues to its crystallography) is possible from the TGA and combustion analysis results. As noted, the TGA results revealed that 85.2% of the bulk mass (i.e., the mass following the surface loss of 2.0 wt.%) is attributable to Mo, whereas the combustion analysis reveals 11.0% of the mass is attributable to N. The combined mass percentages of these two components are only 96.2% and thus, 3.8% of the mass is unaccounted for. The only possible species given the nature of the synthesis and the analysis methods is oxygen. Hydrogen, the only other species present in the reaction, is already ruled out due to its very low mass prevalence in the bulk of the material. Taking the missing mass to be oxygen incorporated in the bulk, the implied material stoichiometry is Mo:N:O = 1:0.88:0.27. Because the ratio of Mo to the sum of N and O is smaller than one, accommodation of such a stoichiometry within the δ-MoN structure, would require the material to host either a large concentration of Mo vacancies (~13%) or a large concentration of N/O interstitials (~15%). The Mo-vacancy model is largely ruled out because the density of such a structure, 8.10 g/cm^3^, would be lower than the measured density of 9.01(1) g/cm^3^. The N/O interstitial model, on the other hand, would yield a material density of 9.35 g/cm^3^. While formally compatible with the measured density if the particles hosted closed porosity, the close-packed δ-MoN structure [3] is not suitable for substantial concentrations of interstitials. We thus attribute the excess oxygen, beyond an atomic ratio of Mo:(N+O) = 1:1, to the presence of a surface oxide layer which is gradually removed over the temperature range from 400 to 700 °C. The upper temperature value is identified on the basis of acceleration in the rate of mass loss at 700 °C, indicating a change in the source of volatile species, and which we specifically attribute to a transition to bulk loss. Between 400 and 700 °C, the mass loss is ~2.2% (relative to the surface-desorbed mass) and corresponds to a surface concentration of oxygen of approximately one atom per 3.5 Å^2^, on the order of the Mo surface site density. After accounting for the mass loss due to surface oxide, the bulk material then must have ~1.6 mass % oxygen to reconcile the TGA and combustion analysis results. In turn, this implies an atomic ration of Mo:(N+O) of 1:1.00 and an overall stoichiometry of MoN_0.89_O_0.11_.

**Figure 6:**
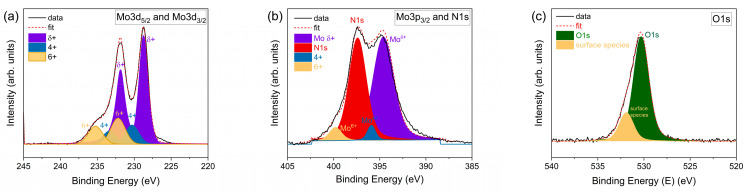
XPS collected in situ at 250 °C of δ-MoO_x_N_y_ produced by the ammonolysis of MoO_2_ in the presence of steam (Table 1, condition 7): (**a**) Mo3d region, (**b**) Mo3p and N1s regions, and (**c**) O1s region. Peak deconvolution was performed in (**a**) using three sets of doublet peaks (3d_5/2_ and 3d_3/2_) corresponding to Mo^δ+^, Mo^4+^, and Mo^6+^; in (**b**) using one N1s peak and three Mo3p_3/2_ peaks corresponding to Mo^δ+^, Mo^4+^, and Mo^6+^; and in (**c**) using one O1s and one surface species. Fit parameters are reported in Table S1.

Additional evidence of oxygen incorporation into δ-MoO_x_N_y_ derives from the XPS results, Figure 6. The spectra were collected at 250 °C (in situ), with the objective of removing surface sorbed species. The O1s spectrum reveals the presence of two types of oxygen species, Figure 6c. The peak at 531.9(1) eV is attributed to loosely bound oxygen, likely H_2_O, whereas the peak at 530.3(1) eV is attributed to tightly bound, bulk-like oxygen reflecting both a surface oxide layer and oxygen within the bulk. Beyond the usual limitation of XPS being dominated by surface chemistry, quantification of the composition is challenged by the overlap of the N1s and Mo3p_3/2_ signals, Figure 6b, as well as the presence of multiple Mo oxidation states, Figure 6a,b. Following peak deconvolution to determine the integrated areas of the various peaks, we find an approximate stoichiometry of Mo:N:O of 1:0.74:0.55 using the sum of the Mo^6+^, Mo^4+^, and Mo^δ+^ 3p_3/2_ peaks, the N1s peak, and O1s (bulk-like) peak (Supplementary Materials Table S1). This differs substantially from the stoichiometric ratio of 1:0.88:0.11 inferred from the TGA, combustion analysis, and PGAA. The high oxygen signal detected by XPS is consistent with the need to implement more aggressive measures than exposure to 250 °C in vacuum to remove the surface oxide layer. Further evidence of the presence of a surface oxide layer under these conditions is provided by comparison of the Mo oxidation state distributions in the Mo3d (doublet 5/2 and 3/2) and Mo3p_3/2_ signals. The former, positioned at lower binding energy, reveals a higher concentration of Mo^6+^ and Mo^4+^ relative to Mo ^δ+^ than the latter. As the electron inelastic mean free path rises approximately linearly with electron energy beyond a value of ~100 eV [31], the Mo3d signal preferentially reflects the composition at the surface. The Mo3p_3/2_ region is dominated by the Mo^δ+^ signal, and we take this species to reflect the state of Mo in the bulk. The peak for this species occurs at 228.7(1) eV, which coincides exactly with the position for Mo^3+^ reported by Choi and Thompson [32] and is consistent with the value of 2.9 that would be expected from a stoichiometry of MoN_0.89_O_0.11_ if N and O were in the oxidation states of −3 and −2, respectively.

Considering the qualitative nature of the XPS chemical analysis, the stoichiometry determined from the other three methods (combustion analysis, TGA, and PGAA) is taken to represent the true chemistry of the δ-MoO_x_N_y_ prepared here. The greatest uncertainty in this case derives from the challenge in differentiating mass loss due to surface desorption and bulk reduction in the TGA measurements. For example, while the mass losses under Ar and under H_2_ at 400 °C are similar, they are not identical, and the possibility of some bulk mass loss at this temperature under H_2_ cannot be entirely ruled out. The uncertainty that can be assigned to the behavior of the surface, moreover, is not readily quantified. Nevertheless, the overall agreement between the various analysis methods suggests that the inferred stoichiometry of MoN_0.89_O_0.11_ is accurate to within ~2 %. This composition is noteworthy in that the N:O ratio is higher than in γ-MoO_x_N_y_ produced by dry ammonolysis at the same reaction temperature of 700 °C (0.75:0.25). Thus, the transformation from γ to δ in the presence of steam (Figure 4a) suggests that γ-MoO_x_N_y_ is metastable, and that steam serves to enhance the kinetics of the transformation to the more stable δ phase, rather than pushing the system away from a condition in which δ is thermodynamically unfavorable.

### 4.2. Electron Microscopy and Spectroscopy

**Figure 7:**
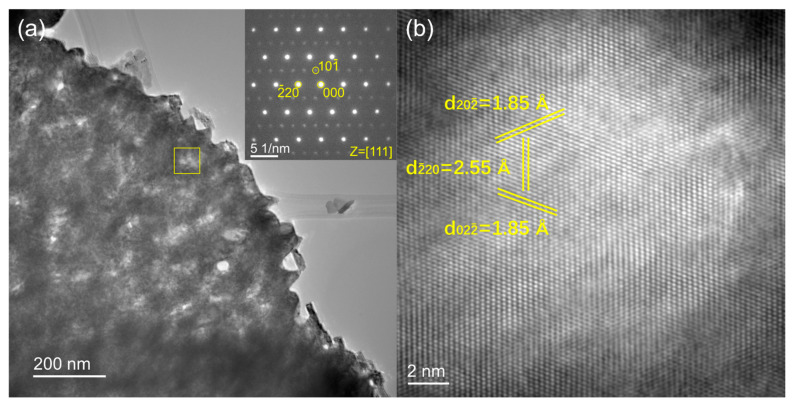
Transmission electron microscopy study of δ-MoO_x_N_y_: (**a**) TEM image with inset showing the SAED pattern along the [111] zone axis; and (**b**) HRTEM image obtained from the highlighted region in (**a**). Selected lattice planes and their corresponding distances are marked in (**b**).

The typical internal structure of δ-MoO_x_N_y_ particles produced by steam-assisted ammonolysis of MoO_2_ is presented in Figure 7a. The selected area diffraction (SAED) pattern, inset in Figure 7a, is consistent with space group P6_3_mc, with the crystal oriented along the [111] zone axis. Additional SAED patterns along different zone axes (Figure S6) confirm the space group as well as the unit cell size. The mottled variation in darkness across the sample image in Figure 7a reflects differences in thickness and porosity of the synthesized particles. A high-resolution TEM image, taken from a thinner area, is shown in Figure 7b. The interplanar *d*-spacings of the (20$\bar{2}$), ($\bar{2}20$), and (02$\bar{2}$) planes identified in the image are 1.85, 2.55, and 1.85 Å, respectively, consistent with the cell parameters obtained from the lab X-ray diffraction analysis (*a* ≈ 5.730 Å and *c* ≈ 5.608 Å). Rather remarkably, the large mesoporous particle visible in Figure 7a is one single crystal, as every region examined across the particle (several micrometers in lateral dimensions) exhibited the identical diffraction pattern, and several of these patterns were obtained using a selected area aperture with an effective diameter up to ~620 nm. This behavior mirrors that of γ-MoO_x_N_y_ obtained by dry ammonolysis of MoO_3_, despite the use of a different precursor and the generation of a different product [7,29]. Topotactic transformation (in which a single crystal of one phase transforms to a single crystal of a different phase) in the reaction of MoO_3_ to form γ-MoO_x_N_y_ is understood to be responsible for the extremely high surface area in the resulting product [5,29]. The present study suggests that the transformation from MoO_2_ to δ-MoO_x_N_y_ is also topotactic in nature, however, the surface area of the product is much lower, 30 vs. 110–170 m^2^/g [27]. The reasons, be they due to differences in the chemistry of the precursor or the chemistry of the product or some other factor, were not further explored. Nevertheless, one factor that can be largely eliminated is a difference in the specific volume associated with Mo in the di- and trioxide precursors as these are rather similar, 65.8 and 67.7 Å^3^, respectively, based on the reported crystal structures [33,34].

**Figure 8:**
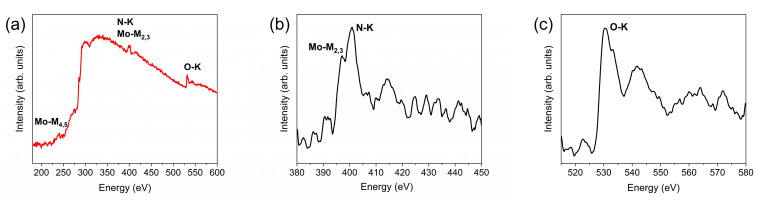
Electron energy loss spectroscopy study of δ-MoO_x_N_y_: (**a**) core-loss EELS recorded with a dispersion of 0.25 eV/ch to resolve the position and shape of the Mo-M_-4,5_, Mo-M_2,3_, N-K, and O-K edges, (**b**) the electron-energy-loss near edge structure (ELNES) of the Mo-M_2,3_ edge, and (**c**) the ENLES of the O-K edge.

The EELS measurements, Figure 8, revealed the presence of oxygen within the bulk of the ≈50 nm thick particles, along with N and Mo. The Mo_M-4,5_, Mo_M2,3_, N_K_, and O_K_ edges can all be observed in the EELS spectrum, Figure 8a. The Mo_M2,3_ and N_K_ edges, at ~400 eV, are strongly overlapped, Figure 8b. The peak positions reported in Table 2 were obtained by fitting Gaussian functions, Figure S7a. The values match closely to what we have obtained previously for two variants of γ-MoO_x_N_y_, though the relative intensity of the N_K_ peak is much stronger in the δ-MoO_x_N_y_ of the present study (comparisons provided in Figures S7 and S8a). A split is evident in the peak of the oxygen ENLES of δ-MoO_x_N_y_. This feature results from the transitions of oxygen s to 2p orbitals hybridized with the Mo 4d orbitals [4,28]. The shape of the spectrum is quite distinct from that of the two variants of γ-MoO_x_N_y_ we studied previously [7] and surprisingly similar to that of MoO_3_ [6], Figure S8b. Elaboration of the details of the electronic interactions that give rise to the features observed in the EELS measurements is beyond the scope of this work.

### 4.3. Neutron and X-Ray Diffraction Structure Refinement

**Figure 9:**
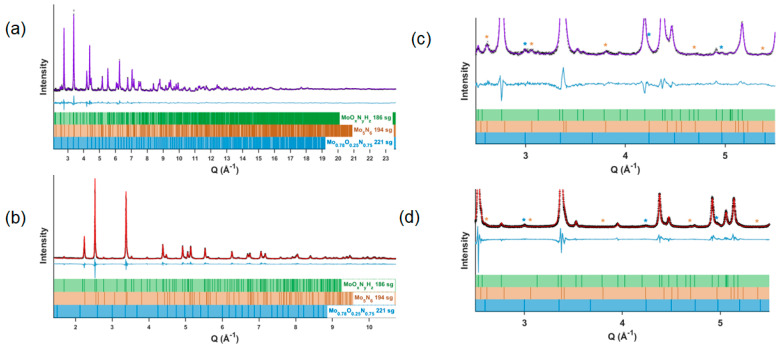
Measured and calculated powder diffraction patterns, the latter from co-refinement of δ-MoO_x_N_y_H_z_ using combined (**a**,**c**) neutron powder diffraction and (**b**,**d**) X-ray synchrotron data. Measured NPD data in (**a**,**c**) are shown as black crosses (×) and calculated patterns as solid purple lines. Measured XRD data in (**b**,**d**) are shown as black plus symbols (+) and calculated patterns as solid red lines. Below each plot, the peak positions for the main phase *(P6_3_mc*, 88.35(9) wt.%, green), the secondary Mo_5_N_6_ phase (*P6_3_/mmc*, 9.71(8) wt.%, brown) and the secondary γ-MoO_x_N_y_ phase (*Pm*$\bar{3}$*m*, 1.94(4) wt.%, blue) are shown as vertical lines. Visible secondary phase peaks that do not overlap with those of the main phase are marked with * in (**c**,**d**). Rietveld refinement residuals: NPD 3.509%, XRD 15.009%, overall wR = 7.830%, and GOF = 1.62.

Using the above insights into the chemical composition of δ-MoO_x_N_y_, particularly the presence of oxygen, structural refinements to simultaneously fit to the time-of-flight neutron powder diffraction (NPD) and X-ray synchrotron (XRD) data were undertaken. A summary of the co-refinement results is provided in Table 3, and the measured and final calculated patterns are presented in Figure 9. As indicated in Figure 9c,d, some peaks in the XRD and ND patterns, particularly the latter, could not be fitted using only the space group *P*6_3_*mc* and lattice parameters of the initially identified δ-MoO_x_N_y_. The peak at *Q* = 3.00 Å^−1^ was readily identified as the (2 0 0) peak of cubic γ-MoO_x_N_y_ (*Pm*$\bar{3}$*m*, *a* = 4.19 Å) [7]. To determine the origin of the remaining unfit peaks, several molybdenum nitride phases of hexagonal symmetry that have been reported in the literature were investigated as either alternatives to the *P*6_3_*mc* phase or as impurities: Mo_5_N_6_ with space group *P*6_3_/*m* (176), *a* = 4.892 Å, *c* = 11.064 Å [30]; Mo_5_N_6_ with space group *P*6_3_/*mmc* (194), *a* = 2.827 Å, *c* = 11.075 Å [35]; δ-MoN with space group *P*6_3_/*mmc* (194), *a* = 2.847 Å, *c* = 11.147 Å [35]; δ_1_-MoN with space group *P*$\bar{6}$*m*_2_ (187), *a* = 2.868 Å, *c* = 2.810 Å [30], and δ_2_-MoN with space group *P*6_3_/*mmc* (194) *a* = 2.86 Å, *c* = 5.69 Å [30]. Structures with space groups *P*6_3_ (173), *P*31*c* (159), and *P*3*m*1 (156), subgroups of space group *P*6_3_*mc* (186), were also explored for describing the main phase. The challenges of differentiating these possible structures, which display only subtle differences, have been explicitly discussed by Ganin et al. [30] Ultimately, the best fits were obtained using the space group *P6_3_mc* (186) for the main δ-MoO_x_N_y_ phase, described as the δ_3_ phase by Ganin et al., [30] and assigning minor peaks to hexagonal Mo_5_N_6_ *P*6_3_/*mmc* (194) [35] and to cubic γ-MoO_x_N_y_ *Pm*$\bar{3}$*m* (221) [7], with refined quantities of 9.71(8) wt.% and 1.94(4) wt.%, respectively. While a search for evidence of secondary phases was not systematically pursued in the TEM, it is noted that the SAED in Figure S6 shows faint peaks that could not be assigned to the main crystal and which may thus reflect the presence of a distinct phase as concluded from the bulk diffraction analysis, though it might also be due to a crystallite of δ-MoO_x_N_y_ with a different orientation. The final refinement of the structure model with these two impurity phases yielded a weighted residual (wR) of 7.830% for the co-refinement, with 3.509% and 15.009% wR obtained for the NPD and XRD, respectively. The refinement statistics were found to be only slightly improved over a model in which the Mo_5_N_6_ phase was assigned the structure reported by Ganin et al. [30] with space group *P*6_3_/*m* (176), Figure S9 and Table S2. Accordingly, the identification here of the structure of Mo_5_N_6_ should not be considered definitive.

The presence of multiple phases creates some difficulty in utilizing the bulk chemical analysis to define the stoichiometry of the main δ-MoO_x_N_y_ phase as each of the three phases can conceivably incorporate oxygen to different extents. In light of the relatively small weight fraction of the Mo_5_N_6_ phase, it was taken to be free of oxygen and to display the ideal Mo:N ratio of 5:6. The stoichiometry of the cubic phase was taken to be Mo_0.78_O_0.25_N_0.75_, as determined in our earlier work which was carried out at the same reaction temperature of the present study [7]. While this chemical formula may not accurately represent the γ phase encountered here, the precise stoichiometry of this very minor phase has little impact on the overall sample composition. With the two impurity phases defined, the site preference of oxygen in the structure of δ-MoO_x_N_y_ was explored. As noted, the measured stoichiometry and density of the δ-MoO_x_N_y_ phase suggested full occupancy on both the Mo and the N sites. Accordingly, refinements were pursued under the constraint of full site occupancies and only allowing the N:O ratio to vary on the two N sites of the δ_3_-MoN structure, one located on a 2b position and the other on a 6c position. Additionally, the N:O ratio was weighted to match the measured chemistry by application of a restraint (weight equivalent of 300 observations) in the refinement. This treatment (with coordinates and displacement parameters of the oxygen and nitrogen atoms on shared sites constrained to be equal) led to 100% nitrogen on the 2b site, which was then taken to be fixed at that value for subsequent analysis. In the later stages, isotropic displacement parameters (U_iso_) were refined for all atoms in δ-MoO_x_N_y_, with the N and O species constrained to have the same U_iso_, whereas for the minor phases, only the weight fractions and lattice parameters were refined. The atomic positions, occupancies and isotropic displacement parameters (U_iso_) of these phases were fixed to the literature values [7,35]. Fourier difference maps of the NPD pattern were used to locate a possible hydrogen position in δ-MoO_x_N_y_, Table 3. The refined occupancy on this 2b site at (1/3, 2/3, 0.575(16)) was 4.8(1.8)%.

The final composition for the main δ-MoO_x_N_y_ phase derived from the co-refinement was MoO_0.108(8)_N_0.892(8)_H_0.012(5)_. In combination with the fact that 9.71(8) wt.% of the sample consists of Mo_5_N_6_ and 1.94(4) wt.% of Mo_0.78_O_0.25_N_0.75_, the overall global composition is MoO_0.105(7)_N_0.905(7)_H_0.011(4)_. The good agreement with the bulk chemical analyses, obtained as MoO_0.11_N_0.89_, is not surprising given the constraints (full site occupancy) and restraints (N:O weighted to 0.98:0.11) used in the treatment of δ-MoO_x_N_y_, the dominant phase. The refined hydrogen content, which was fully unrestrained, agrees with the conclusion that the bulk phases combined contain no more than ~0.02 H per Mo. The detection of γ-MoO_x_N_y_ as a component of the ammonolysis product is consistent with the lab X-ray analysis, in which this phase was also occasionally observed (Figure S2). As the cubic phase forms along the reaction pathway, Figure 4a, occasional incomplete transformation of this phase into the hexagonal phase is not surprising. The phase Mo_5_N_6_ was not detected in the lab X-ray measurements, despite its higher mass fraction. As evident in the diffraction patterns, Figure 9, its presence is apparent from the co-refinement primarily on the basis of several neutron peaks that do not overlap with the main phase and are largely absent in the X-ray measurement, the strongest being the {1 0 1} reflection at *Q* = 2.61 Å (see also Figure S10). Because only limited neutron diffraction experiments were performed, the conditions which favor Mo_5_N_6_ were not established. However, given the many parameters available for tuning the reaction outcome, it is likely that further optimization of steam-assisted ammonolysis of MoO_2_ would yield phase-pure δ-MoN.

**Figure 10:**
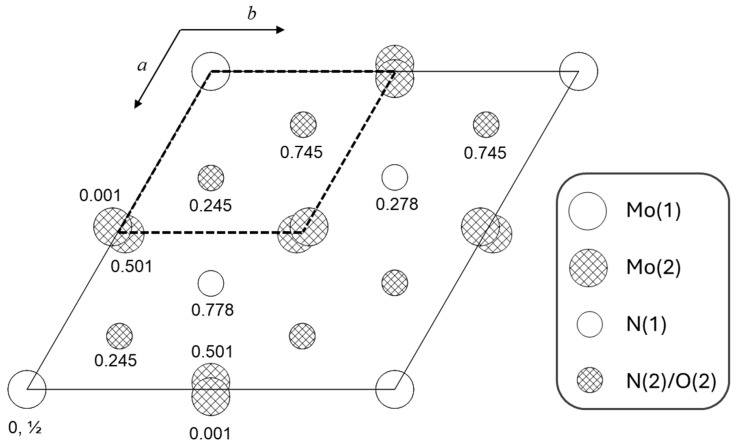
Structure of δ-MoO_x_N_y_ (space group *P6_3_mc*) projected along the *c*-axis. The numbers indicate z-coordinates. The dashed unit cell is that of the NiAs subcell. For clarity, hydrogen atoms are omitted from the figure. They are located at 1/3, 2/3, 0.575, nearly midway between Mo(1) atoms along the z-axis, with a site occupancy of 4.8%.

The structure of δ-MoO_x_N_y_ (isostructural to δ_3_-MoN [30]) is a 2 × 2 × 1 superstructure of the atomic arrangement in NiAs, Figure 10. The Mo(2) and N(2)/O(2) are slightly displaced from the ideal substructure positions. Both types of Mo atoms are octahedrally coordinated by N/O atoms, whereas the N/O atoms are coordinated by Mo in a trigonal prism arrangement. The Mo(1) has only nitrogen from the N(1) site in its coordination polyhedron, Table 4. In contrast, the Mo(2) is coordinated by both N and O, from both N(1) and N(2)/O(2) sites. The lower symmetry at the Mo(2) position enables a wider range of Mo-ligand bond lengths than in the Mo(1) coordination polyhedron, reflected in a larger bond-length distortion factor (*S* = $\left[\frac{\sum_{i}\left|d_{i}-d_{ave}\right|}{d_{ave}}\right]$, where *d_i_* is the length of bond *i* and *d*_ave_ is the average bond length). The average bond length in the Mo(2) coordination polyhedron is slightly shorter than that of the Mo(1) polyhedron, consistent with the presence of oxygen as a ligand to Mo(2). Additionally, the shortest Mo-ligand distance in the structure, 2.1491(17) Å, is between Mo(2) and N(2), providing further evidence that the site of oxygen incorporation (the N(2) site) has been correctly identified. Average Mo-O bond lengths in molybdenum oxides are 1.98 Å in MoO_3_ [34] and 2.01 Å in MoO_2_ [33], whereas the average Mo-N bond length in δ_3_-MoN is substantially longer, 2.17 Å [3], and comparable to the values obtained here. The hydrogen in δ-MoO_x_N_y_ resides approximately ¼*c* above the N(1) position. The site is tetrahedrally coordinated to N(1), the closest ligand, and three N(2)/O(2) species. Three Mo atoms are closer to the hydrogen than the three N(2)/O(2) atoms, but do not lie within the tetrahedral coordination polyhedron. The N(1)-H bond distance of 1.67(9) Å, though longer than the distance in the ammonia molecule (~1.05 Å), is a chemically sensible value.

## 5. Characterization Methods

Standard (ex situ) laboratory X-ray powder diffraction data were collected using Cu Kα radiation at a scan rate of 2.5°/min, with a step size of 0.05° (Ultima IV, Rigaku, Osaka, Japan). Each sample was ground, placed on a zero background SiC holder, and rotated at a speed of 3.1 rad/s during measurement. Both the ex situ and in situ diffraction data were analyzed by Rietveld refinement using GSAS II [36]. The phase fraction, lattice parameters, and crystallite size were allowed to vary in each Rietveld refinement. To account for non-random crystalline orientation, spherical harmonic preferred orientation corrections were applied. Atomic positions and thermal displacements were held fixed. Chebyshev polynomials with 4−6 terms were used to model the background. The standard reference material LaB_6_ was used to determine instrument broadening, and these instrument parameters were applied to all patterns. Scanning electron microscopy (SEM) images were obtained in secondary electron imaging mode using a Hitachi SU8030 (Tokyo, Japan) equipped with a cold field emission source operating at 15 kV and 10 mA. No prior coating was necessary given the conducting nature of the samples. BET (Brunauer–Emmett–Teller) surface area was determined by nitrogen physisorption isotherms measured at liquid nitrogen temperature using a Micromeritics 3Flex instrument (Norcross, GA, USA) and 0.2 g of material. Data were analyzed using the MicroActiv software package (v 4.06). A Micromeritics AccuPyc II 1340 pycnometer (Norcross, GA, USA), which records the sample volume from He displacement, was utilized for determining density. Three measurements of samples 0.27 g in mass were performed.

For transmission electron microscopy (TEM), the sample was lightly ground, sonicated in ethanol, and dropped onto ultrathin carbon-coated Cu grids. Conventional TEM images and selected area electron diffraction (SAED) patterns were obtained using a JEOL Grand ARM 300F (Tokyo, Japan) operated at 300 kV. Scanning transmission electron microscopy (STEM) and STEM/electron energy loss spectroscopy (EELS) were acquired at 200 kV using a Cs-corrected JEOL ARM 200CF (Tokyo, Japan) equipped with Quantum Dual EELS system (Pleasanton, CA, USA). Core- and low-loss EELS were collected using an entrance aperture of 5 mm and energy dispersion of 0.25 eV/channel, which resulted in a 1.75 eV energy resolution determined by the full width at half maximum (FWHM). The energy uncertainty is taken to be approximately 0.6 eV, estimated as 1/3 (or optimistically 1/5) the width of the zero-loss peak (ZLP) [37]. The convergence angle was set to be 20.6 mrad and probe size of ~2 Å. STEM/EELS datasets were collected using Gatan Microscopy Suite^®^ (GMS) version 3.4. Simultaneous acquisitions from multiple channels were synchronized using the Digiscan^®^ 3 system. Final EELS spectra were obtained by averaging the signal across different areas within the sample. The thicknesses were estimated from the ZLP, which showed t/λ values of 0.50 (~50 nm). Data analysis was performed with GMS (v. 3.4 and v. 3.6) and Origin^®^ (v. 9.9). For EELS analysis, core-loss spectra were aligned using a simultaneously acquired low-loss spectrum. Background subtraction was performed on both spectra using a power law *AE^−r^* in the pre-edge energy window from 180 eV to 220 eV. The multiple scattering features in the measurement were removed by Fourier-ratio deconvolution following methods described by Egerton [37]. The MoM_2,3_ and N_K_ edges overlap at around 400 eV, and the features were thus deconvoluted by peak fitting to obtain peak energies. Plotted spectra are shown after alignment, background subtraction, and Fourier-ratio deconvolution.

Qualitative chemical analysis was performed by XPS using a Thermo Scientific ESCALAB 250Xi instrument (Eindhoven, The Netherlands), equipped with an aluminum anode (Al Kα = 1486.6 eV) X-ray source, an electron flood gun, a scanning ion gun, and a heating stage. Samples were prepared by compacting the powders into a disc, 3.2 mm in diameter. Because Ar etching, which is typically used to remove surface species, is known to modify cation oxidation states in metal oxides [38], the contribution of adsorbed gases to the signal (potentially N_2_, H_2_O, NH_3_, H_2_) was minimized by performing measurements at elevated temperature. Once inside the measurement chamber, the sample was slowly heated to 250 °C and held until the vacuum stabilized at ≈1.3 × 10^−5^ Pa, which required approximately 45 min. Following an initial survey scan which revealed the presence of Mo, N, and O in the oxynitride samples, high resolution data were collected in the Mo3d (220 eV to 240 eV), C1s (279 eV to 298 eV), and O1s (520 eV to 545 eV) regions, using a step size of 0.1 eV with a dwell time of 50 ms and integrating over 10 scans. Charging effects were corrected by referencing to the adventitious carbon Cls binding energy at 284.8 eV. The commercial software package Thermo Scientific™ Avantage (v. 5.9) was used for data processing. The instrument energy resolution of 0.10 eV was taken as the uncertainty in the binding energies determined by this analysis. The attributes of the Mo3d XPS peaks were established by fitting doublets with standard constraints (Mo3d_5/2_ and Mo3d_3/2_ with intensity ratio 3/2 and ∆BE ≈ 3.15 eV). Peaks due to three species were resolved: Mo^6+^ at a BE of ≈232.5 eV, Mo^4+^ at a BE of ≈230.1 eV and Mo^d+^ at a BE of ≈228.7, with the assignments based on literature studies of molybdenum oxides [5,28,32,37].

Quantitative and semi-quantitative chemical analyses were performed using a combination of thermogravimetric analysis (TGA), combustion analysis, and prompt gamma activation analysis (PGAA). Uncertainty in these analyses stems from the variable extent of surface oxidation/hydration [39]. To mitigate against this factor, characterization experiments were generally performed within a few days of sample synthesis. TGA measurements were performed under hydrogen to induce complete reduction and thereby reveal the mass of Mo in the material. Data were collected using a Netzsch STA F3, using approximately 70 mg of ground powder. The sample was loaded into a Pt pan, heated to 900 °C under 3% H_2_ (balance Ar) at a rate of 2 °C/min, and held for 3 h. Laboratory XRD was performed after completion of the TGA experiment to confirm, within detection limits, the presence of only Mo metal in the reduction product. The uncertainty in the TGA mass measurements was determined from the manufacturer reported instrument drift (5 mg/h), in combination with the sample mass and the total measurement time.

The nitrogen and hydrogen content were measured by microchemical combustion analysis using the commercial service provider, Midwest Microlabs. The materials were characterized in both the as-synthesized state and after heat-treatment under Ar at 400 °C to remove surface adsorbed species. Desorption under Argon was performed in the same TGA instrument as used for complete reduction. For this, approximately 50 mg of sample was heated to 400 °C at a rate of 5 °C/min and held for 3 h. The combustion analysis was performed by exposing the samples to ultra-pure oxygen at 1000 °C and evaluating the effluent stream to determine the nitrogen and steam content. In principle, the absolute oxygen content can be determined through an analogous pyrolysis approach, but the facility was unable to perform such a measurement. Reported uncertainties in the N and H mass fractions are as provided by Midwest Microlabs.

PGAA measurements were carried out on as-synthesized samples at the cold neutron PGAA instrument at neutron guide D at the NIST Center for Neutron Research following protocols identical to those employed in a recent study of γ-MoO_x_N_y_ [7]. Mass ratios for Mo:N and Mo:H were calculated using count rates from gamma rays measured in sample spectra and element sensitivities (counts s^−1^ mg^−1^) determined from the measurement of appropriate standards. Oxygen has an extremely low prompt gamma-ray cross-section, rendering quantification of this element by PGAA unfeasible.

Neutron powder diffraction data were collected using the POWGEN instrument of the Spallation Neutron Source at Oak Ridge National Laboratory. The powder sample was loaded into a vanadium can, and a diffraction pattern was measured at ambient temperature using the Bank 1 detector. The data were analyzed over the TOF (time-of-flight) range from 6 × 10^3^ μs to 67 × 10^3^ μs, corresponding to a *Q* range from 2.11 Å^−1^ to 23.64 Å^−1^ (*d*-spacing ≈ 0.265 Å to 2.967 Å). High-resolution X-ray synchrotron powder diffraction data were collected at beamline 11-BM of the Advanced Photon Source at Argonne National Laboratory. The measurement was performed at ambient temperature using an X-ray wavelength of 0.457876 Å, with the sample placed in a capillary spun at ≈90 Hz. The data were analyzed from 5.88 ° to 46.00 °in 2θ (*Q* range from 1.41 Å^−1^ to 10.72 Å^−1^, and *d*-spacing range from 0.59 Å to 4.46 Å). The total number of observations was 3019 for NPD and 40,182 for XRD. The background in the NPD pattern was fitted with an 8th-degree Chebyschev polynomial, whereas for the XRD pattern, a 5th-degree Chebyschev polynomial was used. In addition to crystal structure parameters, as described above, scale factors, sample displacement perpendicular to the beam, uniaxial crystallite size and microstrain parameters were refined. Hydrogen, which is entirely invisible to X-rays (and electrons) in the presence of Mo, can be detected by NPD due to its negative scattering length of −3.7390 fm. A difference Fourier map using the NPD data was computed to identify possible positions of hydrogen atoms. Because the main hexagonal structure (sg 186) is polar and lacks a unique origin along z, it is essential to define the origin by fixing the z coordinate of one of the atoms. Here, Mo(1) was chosen to have z = 0. All analysis was performed using the General Structure Analysis System II (GSAS-II) package [36]. The reported uncertainties are the statistical uncertainties obtained from the GSAS refinements and, for derived quantities, from the numerical propagation of such uncertainties.

## 6. Summary and Conclusions

In this work, the role of precursor phase, gas phase chemistry, and temperature profile on the ammonolysis reaction of NH_3_ with molybdenum oxides was explored with the aim of producing the computationally predicted MoON material, of interest for its ordered oxygen and nitrogen arrangement leading to the potential to display a Peierls metal-insulator transition. Three precursors—MoO_2_, MoO_3_, and H_2_MoO_5_, along with four different gas atmospheres—ranging from dry to 2.5% humidity, and two different heating profiles were examined, for a total of nine different ammonolysis conditions. Both the choice of precursor and the presence of steam in the reaction gas atmosphere had dramatic influences on the reaction outcome. Formation of δ-MoO_x_N_y_ over γ-MoO_x_N_y_ was favored in the sequence MoO_2_ > MoO_3_ > H_2_MoO_5_, whereas the presence of steam favored δ-MoO_x_N_y_ over γ-MoO_x_N_y_ for all precursors. A high phase fraction (approaching 100%) of δ-MoO_x_N_y_ was produced when using MoO_2_ as the precursor and 2% steam. Significantly, under no condition was the computationally predicted phase MoON observed. Increasing the steam content in the reactant gas stream to 2.5% during ammonolysis of MoO_3_ resulted in the formation of MoO_2_ rather than transforming the product to an oxygen-rich oxynitride. In situ XRD revealed that γ-MoO_x_N_y_ and δ-MoO_x_N_y_ appeared simultaneously, whether under anhydrous or humidified ammonolysis, and that steam enhanced the rate at which γ-MoO_x_N_y_ transformed to δ-MoO_x_N_y_ at 700 °C, without suppressing or bypassing the formation of γ-MoO_x_N_y_. On the basis of the parameter space exploration, the following synthesis protocol was established for the preparation of δ-MoO_x_N_y_: employ MoO_2_ as the precursor and *p*H_2_O = 0.020 atm (balance NH_3_) as the reactant gas; utilize a mass normalized gas flow rate of 800 sccm/g; heat from ambient temperature to 700 °C at 3 °C/min; hold for 12 h at 700 °C. Utilization of steam in the reactant gas stream appears essential to the preparation of δ-MoO_x_N_y_.

The resultant δ-MoO_x_N_y_ material was found to have a specific surface area of 30.1(6) m^2^/g, moderate surface porosity, and a bulk composition of MoO_0.108(8)_N_0.892(8)_H_0.012(5)_ with an oxide surface layer composed of approximately one O atom per Mo surface site. Furthermore, δ-MoO_x_N_y_ particles retained the micron-scale dimensions of the MoO_2_ precursor, but were formed of nanoscale sub-crystallites with identical orientation, typical of a topotactic transformation. Neutron and synchrotron X-ray diffraction co-refinement identified the presence of impurity phases not detectable by laboratory XRD, specifically, 9.71(8) wt.% Mo_5_N_6_ and 1.94(4) wt.% γ-MoO_x_N_y_, with the neutron diffraction also revealing the presence of a small amount of hydrogen in the bulk of the material. Refinement of the structure of the δ-MoO_x_N_y_ phase showed it to be space group *P6_3_mc* (isostructural to δ_3_-MoN [30]), with the O atoms located only on the N(2) site and hydrogen located 1.67(9) Å from the N(1) position. The Mo(1) species is coordinated by nitrogen only from the N(1) site, whereas the Mo(2) is coordinated by nearest neighbors on both the nitrogen-only N(1) site and the mixed-occupancy N(2) site. The shortest Mo-X bond in the structure is between Mo(2) and N(2), consistent with the incorporation of oxygen at the N(2) site. Detection of the preferential ordering of N and O in δ-MoO_x_N_y_ was only possible via the use of neutron diffraction. We have similarly observed partial O/N ordering in the cubic γ-MoO_x_N_y_ phases [7], again, using neutron diffraction. The relatively limited study of nitrides by neutron diffraction methods raises the possibility that oxygen incorporation and O/N ordering may be more common in such materials than appreciated.

The surprising influence of steam on the reaction outcome in the ammonolysis of Mo bearing oxides underscores the challenges of designing reaction pathways to obtain targeted outcomes. The fact that steam induces the transformation from γ-MoO_x_N_y_, likely the γ’ phase with x/(x + y)~0.25, to δ-MoO_x_N_y_ with x/(x + y)~0.1, suggests a catalytic rather than thermodynamic influence of H_2_O, the origin of which awaits further study. In light of the observation here that increasing the H_2_O partial pressure in the reaction favored the formation of MoO_2_, future efforts to produce stoichiometric MoON may benefit from utilizing lower ammonolysis temperatures, at which reduction is less favorable. Decreased synthesis temperatures also typically favor ordered arrangements, a likely prerequisite for the emergence of a Peierls transition in MoON, though potentially detrimental hydrogen incorporation may also be favored.

## Figures and Tables

**Table 1:** Synthesis conditions utilized for studying the influence of precursor, gas composition, and heating profile during ammonolysis of selected Mo-bearing precursors. The resulting mass % of the hexagonal δ-MoO_x_N_y_ phase is also indicated. The flow rate is reported in units of standard cubic centimeters per minute (sccm).

Condition	Experiment Description	*p*NH_3_, atm	*p*Ar, atm	*p*H_2_O, atm	Flow Rate, sccm	% δ-MoO_x_N_y_
1	MoO_3_—dry	1.0	0	0	100	2
2	MoO_2_—dry	1.0	0min	0	100	29
3	H_2_MoO_5_—dry	1.0	0	0	100	0
4	MoO_3_—humid (1.5%)	0.493	0.492	0.015	203	24
5	MoO_3_—humid (2%)	0.328	0.652	0.020	305	97
6	MoO_3_—humid (2.5%)	0.189	0.786	0.025	528	67
7	MoO_2_—humid (2%)	0.328	0.652	0.020	305	100
8	H_2_MoO_5_—humid (2%)	0.328	0.652	0.020	305	30
9	MoO_3_—humid (2%)—slow ramp *	0.328	0.652	0.020	305	36

* Temperature profile: RT to 350 °C at 5 °C/min; 350 to 500 °C at 0.6 °C/min; 500 to 700 °C at 3 °C/min, held for 12 h. All other syntheses are performed using RT to 700 °C at 3 °C/min, held for 12 h.

**Table 2:** Summary of physico-chemical properties of δ-MoO_x_N_y_ produced by the ammonolysis of MoO_2_ in the presence of steam (Table 1, condition 7). Estimated uncertainty in the final digit(s) of the reported values are provided in parentheses.

Thermogravimetric Analysis	
Mass loss under Ar at 400 °C (surface)	2.00(1) wt.%
Mass loss under H_2_ at 400 °C (surface)	2.66(1) wt.%
Mass loss under H_2_ at 700 °C (surface)	4.15(3) wt.%
Mass loss under H_2_ at 900 °C	16.53(6) wt.%
Mass loss under H_2_ from 700 to 900 °C (bulk)	17.25(6) wt.% relative to mass at 700 °C
**Combustion Analysis**	
Absolute N content, as-synthesized	11.7(3) mass %
Absolute N content, surface-desorbed	11.0(3) mass %
Absolute H content, as-synthesized	0.4(3) mass %
Absolute H content, surface-desorbed	0.2(3) mass %
**Prompt Gamma Activation Analysis**	
Mo:N atomic ratio	1.03(3)
Mo:H atomic ratio	4.05(3)
**BET Surface Area**	30.1(6) m^2^/g
**Pycnometry** (measured density)	9.01(1) g/cm^3^
**X-ray Photoelectron Spectroscopy**	
Mo^d+^ binding energy	228.7(1) eV
N:O	1.34
**Electron Energy Loss Spectroscopy**	
Mo edge peak	396.7(6) eV
N edge peak	400.9(6) eV

**Table 3:** Summary of the Rietveld co-refinement of neutron time-of-flight powder diffraction and synchrotron X-ray diffraction data collected from δ-MoO_x_N_y_H_z_ produced by the ammonolysis of MoO_2_ in the presence of steam (Table 1, condition 7). The estimated uncertainty in the final digit(s) of refined values is provided in parentheses. The crystallographic information for the secondary phases was taken from the literature [7,35].

Space group	*P*6_3_*mc* (186)				
Lattice parameters (Å)	*a* = *b* = 5.73835 (13) |*c* = 5.61840 (7)				
Composition	MoO_0.108(8)_N_0.892(8)_H_0.012(5)_				
Density (g/cm^3^)	9.135				
**Site|Element**	**x**	**y**	**z**	**Occupancy**	**Uiso, × 10^2^ Å^2^**
Site 2(a), 3*m* | Mo(1)	0	0	0.0 (fixed)	1 (fixed)	0.14(2)
Site 6(c), *m* | Mo(2)	0.48875(4)	0.51125(4)	0.0010(5)	1 (fixed)	0.038(6)
Site 2(b), 3*m* | N(1)	1/3	2/3	0.2779(7)	1 (fixed)	0.259(4)
Site 6(c), *m* | N(2) + O(2)	0.1676(2)	0.8324(2)	0.7453(7)	0.856(10) N	0.259(4)
(net occupancy fixed to 1)				0.144(10) O	
Site 2(b), *m* | H(1)	1/3	2/3	0.575(16)	0.048(18)	1.00 (fixed)
Secondary phase Mo_5_N_6_ *P*6_3_*/mmc* (194) [35]		9.71(8) wt.%			
Lattice parameters (Å)		*a* = *b* = 2.8394(3) | *c* = 11.1563(11)			
Density (g/cm^3^)		8.148			
Secondary phase Mo_0.78_O_0.25_N_0.75_ *Pm*$\bar{3}$*m* (221) [7]		1.94(4) wt.%			
Lattice parameters (Å)		*a* = *b* = *c* = 4.1886(6) Å			
Density (g/cm^3^)		8.031			
Overall sample composition		MoO_0.105(7)_N_0.905(7)_H_0.011(4)_			
		**Residual wR (%)**			
Neutron		3.509%, 3019 observations			
X-ray		15.009%, 40182 observations			
Combined		7.830%			
**Goodness of Fit**		1.62			

**Table 4:** Coordination environment of Mo atoms in δ-MoO_x_N_y_H_z_ produced by the ammonolysis of MoO_2_ in the presence of steam (Table 1, condition 7). *S* is the bond-length distortion factor, a measure of the octahedral distortion.

Mo(1)	Distance, Å	Mo(2)	Distance, Å
N(2) × 3	2.197(3)	N(1)	2.192(2)
N(2) × 3	2.162(3)	N(1)	2.1676(17)
		N(2) × 2	2.1933(16)
		N(2) × 2	2.1491(17)
*S*	0.047	*S*	0.052

## Data Availability

The original contributions presented in this study are included in the article/Supplementary Material. Further inquiries can be directed to the corresponding authors.

## Supplementary Materials

The following supporting information can be downloaded at: https://www.mdpi.com/article/10.3390/ma18102340/s1, Figure S1: Lab XRD patterns of in-house synthesized H_2_MoO_5_, commercial MoO_2_, and commercial MoO_3_, as labeled. Data collected using Cu Kα radiation (λ = 1.5406 Å). Figure S2: Lab XRD of the product of several MoO_2_ ammonolysis reactions using the procedures indicated in condition 7 of Table 1 (main text). Data collected using Cu Kα radiation (λ = 1.5406 Å). Peak positions for the reference compounds δ-MoN, γ-Mo_2_N, and Mo_5_N_6_ are shown for comparison. While δ-MoO_x_N_y_ was always the majority phase, occasionally, minor quantities of γ-MoO_x_N_y_ were observed. Samples with secondary phases were not used for further characterization. Figure S3: Contour plot of diffraction patterns from in situ XRD measurements upon the ammonolysis of MoO_2_ under hydrous conditions, using 2.04% steam (see main text Figure 4a for phase evolution analysis). Data collected using Cu Kα radiation (λ = 1.5406 Å). Figure S4: Thermogravimetric profiles of δ-MoO_x_N_y_ produced by the ammonolysis of MoO_2_ in the presence of steam (main text Table 1, condition 7) under Ar and under 3% H_2_ (balance Ar). Under Ar, mass loss is attributed to the removal of surface sorbed species. Under H_2_, with the measurement carried out to higher temperature, the material undergoes complete reduction to yield metallic Mo. Figure S5: PGAA spectra in the (a) Mo, (b) H, and (c) N regions obtained from δ-MoO_x_N_y_H_z_ produced by the ammonolysis of MoO_2_ in the presence of steam (main text Table 1, condition 7). Figure S6: SAED patterns collected along the (a) [221] zone and (b) [331] axes of a crystallite of δ-MoO_x_N_y_ produced by the ammonolysis of MoO_2_ in the presence of steam (main text Table 1, condition 7). Figure S7: ELNES of Mo-M_2,3_ edges of molybdenum oxynitrides obtained by ammonolysis: (a) δ-MoO_x_N_y_ (this work, condition 7 of Table 1, main text) (b) γ’-MoO_x_N_y_, and (c) γ”-MoO_x_N_y_. Results in (b) and (c) are taken from our previous work [7]. Gaussian functions were used to fit the peaks of molybdenum and nitrogen. Figure S8: Electron energy loss spectroscopy study of Mo bearing compounds: (a) EELS of δ-MoO_x_N_y_ (this work, condition 7 of Table 1, main text), γ’-MoO_x_N_y_ [7], and γ”-MoO_x_N_y_ [7]; and (b) ELNES of O-K edges of compounds in (a) and of oxygen gas [37,40], MoO_3_ [41], and MoO_2_ [41]. Figure S9: Measured and calculated powder diffraction patterns, the latter from co-refinement of δ-MoO_x_N_y_H_z_ using combined (a,c) neutron powder diffraction and (b,d) X-ray synchrotron data. Measured NPD data in (a,c) are shown as black crosses (×) and calculated patterns as solid purple lines. Measured XRD data in (b,d) are shown as black plus symbols (+) and calculated patterns as solid red lines. Below each plot, the peak positions for the main phase (*P6_3_mc*, 89.19(9) wt.%, green), the secondary Mo_5_N_6_ phase (*P6_3_/m*, 9.07(8) wt.%, brown) and the secondary γ-MoO_x_N_y_ phase (*Pm*$\bar{3}$*m*, 1.84(4) wt.%, blue) are shown as vertical lines. Visible secondary phase peaks that do not overlap with those of the main phase are marked with * in (c,d). Rietveld refinement residuals: NPD 3.588%, XRD 14.966%, overall wR = 7.837%, and GOF = 1.62. The fit differs from the treatment in Figure 9 (main text) in that the impurity phase Mo_5_N_6_ is modeled using a structure with a different space group. Figure S10: Simulated powder diffraction patterns of δ-MoO_x_N_y_ (space group *P6_3_mc*, 186) and Mo_5_N_6_ phase (space group *P6_3_/mmc*, 194) using the radiation sources and instrument parameters for (a) POWGEN neutron TOF, (b) 11-BM X-ray synchrotron, and (c) the laboratory X-ray diffractometer employed in this study. The intensities of the phases have been normalized for all patterns. The most intense peaks of Mo_5_N_6_ overlap strongly with those of δ-MoO_x_N_y_. In the NPD patterns, the presence of Mo_5_N_6_ can be recognized by the presence of peaks indicated with asterisks. In the XRD patterns, the presence of Mo_5_N_6_ can only be recognized by the presence of shoulders on the main peaks, requiring high resolution measurements. Treatments of Mo_5_N_6_ in the alternative structure with space group symmetry *P6_3_/m* (176) give essentially identical results. Table S1: Features of the Mo3d, Mo3p-N1s, and O1s peaks detected by thermal XPS (250 °C) in δ-MoO_x_N_y_ produced by the ammonolysis of MoO_2_ in the presence of steam (main text Table 1, condition 7). Table S2: Summary of the Rietveld co-refinement of neutron time-of-flight powder diffraction and synchrotron X-ray diffraction data collected from δ-MoO_x_N_y_H_z_ produced by the ammonolysis of MoO_2_ in the presence of steam (Table 1, condition 7). The estimated uncertainty in the final digit(s) of refined values is provided in parentheses. The crystallographic information for the secondary phases was taken from the literature [7,30].

## Abbreviations

The following abbreviations are used in this manuscript:

BET	Brunauer–Emmett–Teller
EELS	Electron Energy Loss Spectroscopy
ENLES	Electron-Energy-Loss Near Edge Structure
FWHM	Full Width at Half Maximum
GMS	Gatan Microscopy Suite
GSAS	General Structure Analysis System
NPD	Neutron Powder Diffraction
PGAA	Prompt Gamma Activation Analysis
SAED	Selected Area Electron Diffraction
STEM	Scanning Transmission Electron Microscopy
TEM	Transmission Electron Microscopy
TGA	Thermogravimetric Analysis
TOF	Time-of-Flight
XPS	X-Ray Photoemission Spectroscopy
XRD	X-Ray Diffraction
ZLP	Zero-Loss Peak

## References

[1] Fuertes A. (2018). Synthetic approaches in oxynitride chemistry. Prog. Solid State Chem..

[2] Choi J.-G., Curl R.L., Thompson L.T. (1994). Molybdenum nitride catalysts: I. Influence of the synthesis factors on structural properties. J. Catal..

[3] Bull C.L., McMillan P.F., Soignard E., Leinenweber K. (2004). Determination of the crystal structure of δ-MoN by neutron diffraction. J. Solid State Chem..

[4] Jaggers C.H., Michaels J.N., Stacy A.M. (1990). Preparation of High-Surface-Area Transition-Metal Nitrides: Mo_2_N and MoN. Chem. Mater..

[5] Volpe L., Boudart M. (1985). Compounds of molybdenum and tungsten with high specific surface area: I. Nitrides. J. Solid State Chem..

[6] Song X., Yi W., Li J., Kong Q., Bai H., Xi G. (2021). Selective Preparation of Mo_2_N and MoN with High Surface Area for Flexible SERS Sensing. Nano Lett..

[7] Pandey S.A., Zhang C., Ibrahim D.H., Goldfine E.A., Wenderott J.K., dos Reis R., Paul R.L., Spanopoulos I., Kanatzidis M., Bedzyk M.J. (2021). Hidden Complexity in the Chemistry of Ammonolysis-Derived “γ-Mo_2_N”: An Overlooked Oxynitride Hydride. Chem. Mater..

[8] Gouin X., Marchand R., L’Haridon P., Laurent Y. (1994). Action de l’ammoniac sur l’oxyde de molybdene MoO_3_. Caractérisation physico-chimique de la phase oxynitrure de type Mo_2_N γ. J. Solid State Chem..

[9] Lyutaya M.D. (1979). Formation of nitrides of group 6 transition metals of the Periodic system. Poroshkovaya Metall. (Kiev).

[10] Miga K., Stanczyk K., Sayag C., Brodzki D., Djéga-Mariadassou G. (1999). Bifunctional Behavior of Bulk MoO_x_N_y_ and Nitrided Supported NiMo Catalyst in Hydrodenitrogenation of Indole. J. Catal..

[11] Sayag C., Bugli G., Havil P., Djéga-Mariadassou G. (1997). Surface Studies of Passivated Molybdenum Oxynitride. J. Catal..

[12] Kreider M.E., Stevens M.B., Liu Y., Patel A.M., Statt M.J., Gibbons B.M., Gallo A., Ben-Naim M., Mehta A., Davis R.C. (2020). Nitride or Oxynitride? Elucidating the Composition–Activity Relationships in Molybdenum Nitride Electrocatalysts for the Oxygen Reduction Reaction. Chem. Mater..

[13] de Respinis M., Fravventura M., Abdi F.F., Schreuders H., Savenije T.J., Smith W.A., Dam B., van de Krol R. (2015). Oxynitrogenography: Controlled Synthesis of Single-Phase Tantalum Oxynitride Photoabsorbers. Chem. Mater..

[14] Orhan E., Tessier F., Marchand R. (2002). Synthesis and energetics of yellow TaON. Solid State Sci..

[15] Szymanski N.J., Walters L.N., Puggioni D., Rondinelli J.M. (2019). Design of Heteroanionic MoON Exhibiting a Peierls Metal-Insulator Transition. Phys. Rev. Lett..

[16] O’Hara A., Demkov A.A. (2015). Nature of the metal-insulator transition in NbO_2_. Phys. Rev. B.

[17] Griesemer S.D., Baldassarri B., Zhu R., Shen J., Pal K., Park C.W., Wolverton C. (2025). Wide-ranging predictions of new stable compounds powered by recommendation engines. Sci. Adv..

[18] Shen J.H., Hegde V.I., He J.G., Xia Y., Wolverton C. (2021). High-Throughput Computational Discovery of Ternary Mixed-Anion Oxypnictides. Chem. Mater..

[19] Zakutayev A., Bauers S.R., Lany S. (2022). Experimental Synthesis of Theoretically Predicted Multivalent Ternary Nitride Materials. Chem. Mater..

[20] Sharan A., Lany S. (2021). Computational discovery of stable and metastable ternary oxynitrides. J. Chem. Phys..

[21] Michie C.W., Claridge J.B., Clarke S.J., Rosseinsky M.J. (2003). Zr_4_O_5_N_2_—Intergrowth of Fluorite and Bixbyite Anion Layers Formed by Coupled Site-Selective Anion and Vacancy Ordering. Chem. Mater..

[22] Füglein E., Lerch M., Hock R. (1997). Über Kristallstruktur und Hochtemperaturverhalten von Zr_2_ON_2_. Z. Für Anorg. Und Allg. Chem..

[23] Clarke S.J., Michie C.W., Rosseinsky M.J. (1999). Structure of Zr_2_ON_2_ by Neutron Powder Diffraction: The Absence of Nitride–Oxide Ordering. J. Solid State Chem..

[24] Wang C.-H., Kennedy B.J., Menezes de Oliveira A.L., Polt J., Knight K.S. (2017). The impact of anion ordering on octahedra distortion and phase transitions in SrTaO_2_N and BaTaO_2_N. Acta Crystallogr. Sect. B.

[25] Brauer G., Weidlein J.R. (1965). Synthesis and Properties of Tantalum Oxide Nitride TaON. Angew. Chem.-Int. Edit..

[26] Marchand R., Gouin X., Tessier F., Laurent Y., Oyama S.T. (1996). New routes to molybdenum nitrides and oxynitrides: Preparation and characterization of new phases. The Chemistry of Transition Metal Carbides and Nitrides.

[27] Goldfine E.A., Wenderott J.K., Sweers M.E., Pandey S., Seitz L.C., Bedzyk M.J., Haile S.M. (2022). Molybdenum Oxide Precursors that Promote the Low-Temperature Formation of High-Surface-Area Cubic Molybdenum (Oxy)nitride. Inorg. Chem..

[28] Panda R.N., Kaskel S. (2006). Synthesis and characterization of high surface area molybdenum nitride. J. Mater. Sci..

[29] Zhang C., Goldfine E.A., He K., Wenderott J.K., Pandey S.A., dos Reis R., Shen J.H., Wolverton C., Bedzyk M.J., Poeppelmeier K.R. (2024). Elucidating the Reaction Pathway in the Ammonolysis of MoO_3_ via In Situ Powder X-ray Diffraction and Transmission Electron Microscopy. Chem. Mater..

[30] Ganin A.Y., Kienle L., Vajenine G.V. (2006). Synthesis and characterisation of hexagonal molybdenum nitrides. J. Solid State Chem..

[31] Powell C.J. (2020). Practical guide for inelastic mean free paths, effective attenuation lengths, mean escape depths, and information depths in x-ray photoelectron spectroscopy. J. Vac. Sci. Technol. A.

[32] Choi J.G., Thompson L.T. (1996). XPS study of as-prepared and reduced molybdenum oxides. Appl. Surf. Sci..

[33] Bolzan A., Kennedy B., Howard C. (1995). Neutron Powder Diffraction Study of Molybdenum and Tungsten Dioxides. Aust. J. Chem..

[34] Leisegang T., Levin A.A., Walter J., Meyer D.C. (2005). In situ X-ray analysis of MoO3 reduction. Cryst. Res. Technol..

[35] Cao B., Neuefeind J.C., Adzic R.R., Khalifah P.G. (2015). Molybdenum nitrides as oxygen reduction reaction catalysts: Structural and electrochemical studies. Inorg. Chem..

[36] Toby B.H., Von Dreele R.B. (2013). GSAS-II: The genesis of a modern open-source all purpose crystallography software package. J. Appl. Crystallogr..

[37] Egerton R.F. (2011). Electron Energy-Loss Spectroscopy in the Electron Microscope.

[38] Chuang T.J., Brundle C.R., Wandelt K. (1978). X-ray Photoelectron-Spectroscopy Study of Chemical Changes in Oxide and Hydroxide Surfaces Induced by Ar^+^ Ion-Bombardment. Thin Solid Film..

[39] Ruan D., Lin R., Jiang K., Yu X., Zhu Y., Fu Y., Wang Z., Yan H., Mai W. (2017). High-Performance Porous Molybdenum Oxynitride Based Fiber Supercapacitors. ACS Appl. Mater Interfaces.

[40] Sing M., Neudert R., von Lips H., Golden M.S., Knupfer M., Fink J., Claessen R., Mücke J., Schmitt H. (1999). The electronic structure of metallic K_0.3_MoO_3_ and insulating MoO_3_ from high-energy spectroscopy. Phys. Rev. B Condens. Matter Mater. Phys..

[41] Lajaunie L., Boucher F., Dessapt R., Moreau P. (2015). Quantitative use of electron energy-loss spectroscopy Mo-M_2,3_ edges for the study of molybdenum oxides. Ultramicroscopy.

