# Supplementary material for: Steam-Assisted Ammonolysis of MoO2 as a Synthetic Pathway to Oxygenated δ-MoN
Source: Materials (Basel). 2025 May 17;18(10):2340. doi: 10.3390/ma18102340 (PMC12113219; doi:10.3390/ma18102340)
Supplement: Supplementary file 1 [file materials-18-02340-s001.zip › materials-3590588-supplementary.pdf]

Supplementary Information

Steam-assisted ammonolysis of MoO_2 as a synthetic pathway to oxygenated δ-MoN

Shobhit Pandey [1], Elise A. Goldfine [1], Shriya Sinha [2], Chi Zhang [1], Jill K. Wenderott [1], Lucien Kaczmarczyk [2], Ksawery Dabrowiecki [2], Vinayak P. Dravid [1], Gabriela B. González [2,*] and Sossina M. Haile [1,*]

1 Materials Science and Engineering, Northwestern University, Evanston, IL 60208, USA; shobhitpandey2015@gmail.com or shobhitpandey2015@u.northwestern.edu (S.P.); elisegoldfine2@gmail.com or elisegoldfine@u.northwestern.edu (E.A.G.); zhangchivicky@gmail.com or chizhang@u.northwestern.edu (C.Z.); jill.wenderott@drexel.edu (J.K.W.); v-dravid@northwestern.edu (V.P.D.)

2 Physics and Astrophysics, DePaul University, Chicago, IL 60614, USA; shriyasi@umich.edu (S.S.); lucienvkacz@gmail.com (L.K.); kdabrowi@depaul.edu (K.D.)

* Corresponding authors: ggonza18@depaul.edu (G.B.G.) sossina.haile@northwestern.edu (S.M.H.)

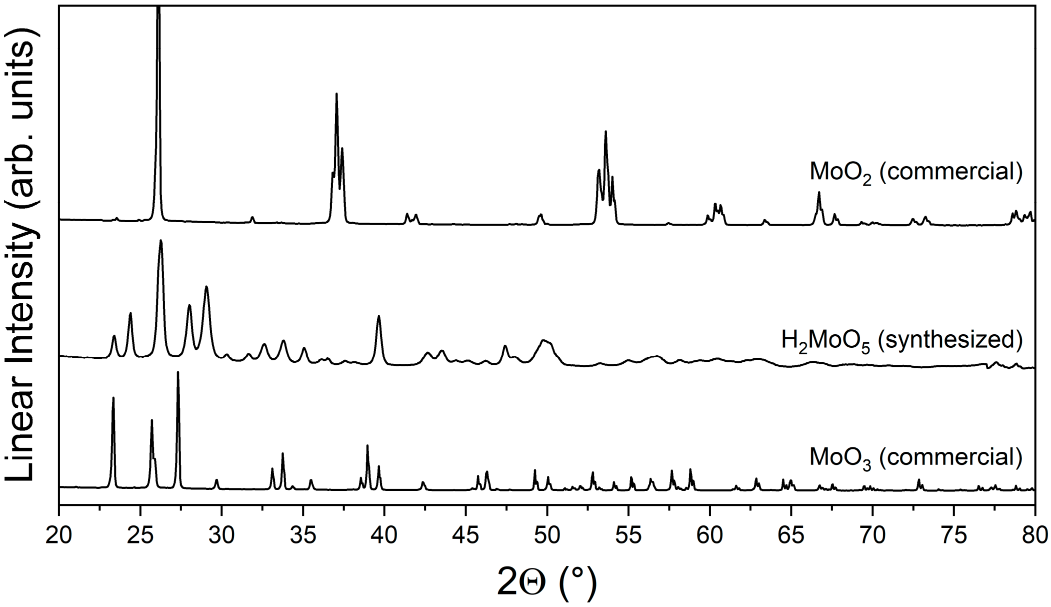

Figure S1: Lab XRD patterns of in-house synthesized H_2MoO_5, commercial MoO_2, and commercial MoO_3, as labeled. Data collected using Cu Kα radiation (λ = 1.5406 Å).

In a typical synthesis of H_2MoO_5, 100 ml of 30% wt. H_2O_2 solution was heated to 40 °C under constant stirring on a magnetic stirrer, to which 2 g MoO_3 (Sigma Aldrich, 99.99%) was added slowly in several parts under constant stirring. The solution was heated and maintained at ~55 °C under constant stirring overnight. During the process, the initial suspension changes color from white to light green to bright yellow solution. Finally, the solution was naturally evaporated off by storing it for 48 hrs in a fume hood. The resulting bright yellow powder (H_2MoO_5) was collected and dried in an oven at 80 °C overnight in air.

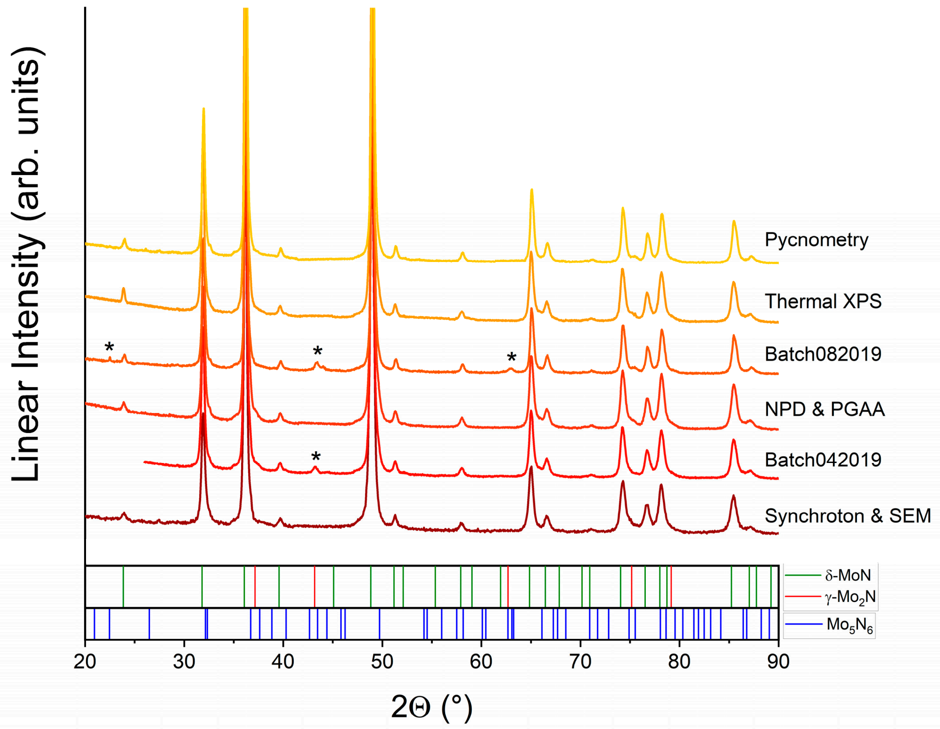

Figure S2: Lab XRD of the product of several MoO_2 ammonolysis reactions using the procedures indicated in condition 7 of Table 1 (main text). Data collected using Cu Kα radiation (λ = 1.5406 Å). Peak positions for the reference compounds δ-MoN, γ-Mo_2N and Mo_5N_6 are shown for comparison. While δ-MoO_xN_y was always the majority phase, occasionally, minor quantities of γ-MoO_xN_y were observed. Samples with secondary phases were not used for further characterization.

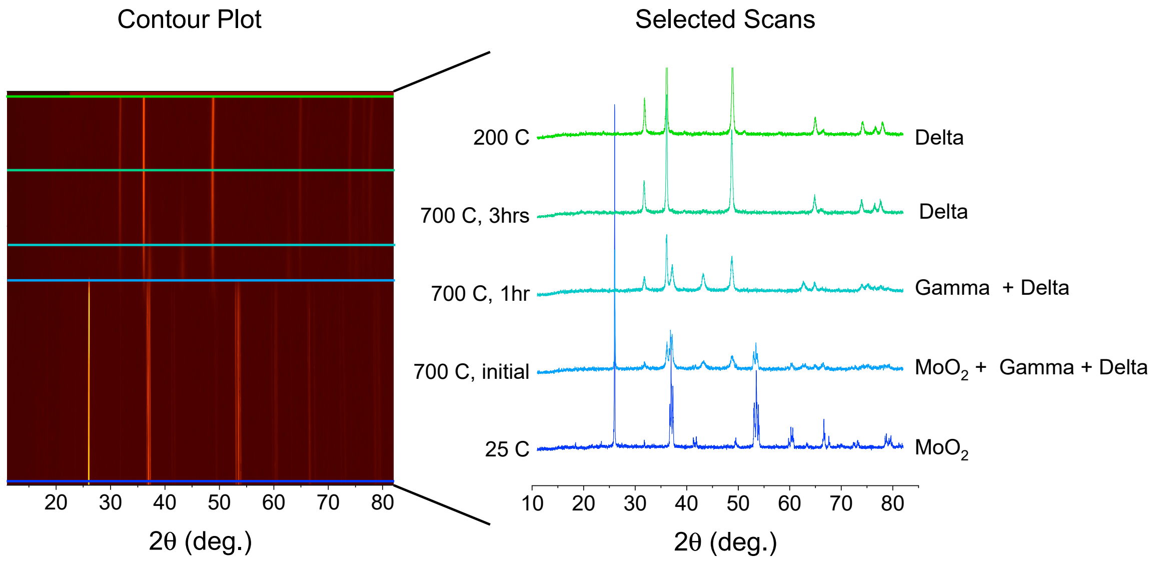

Figure S3: Contour plot of diffraction patterns from in situ XRD measurements upon ammonolysis of MoO_2 under hydrous conditions, using 2.04 % steam (see main text Figure 4a for phase evolution analysis). Data collected using Cu Kα radiation (λ = 1.5406 Å).

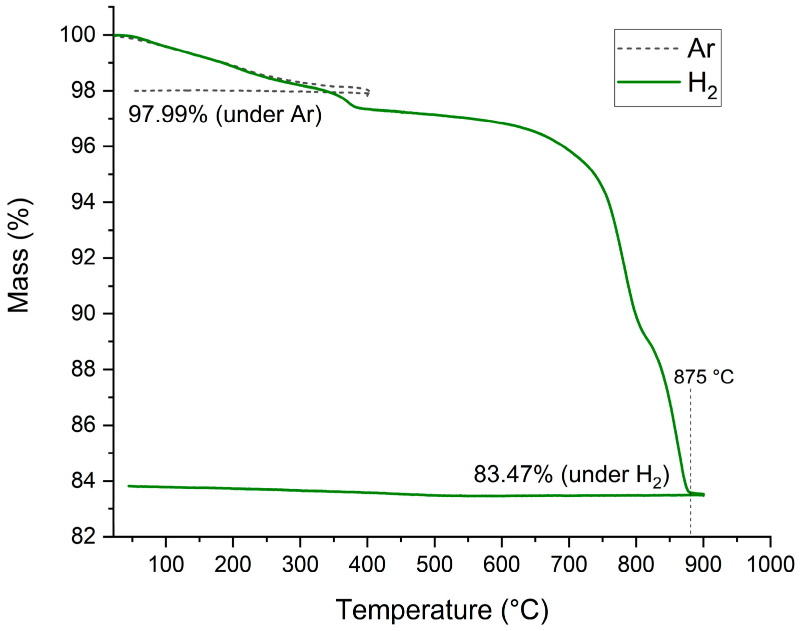

Figure S4: Thermogravimetric profiles of δ-MoO_xN_y produced by the ammonolysis of MoO_2 in the presence of steam (main text Table 1, condition 7) under Ar and under 3% H_2 (balance Ar). Under Ar, mass loss is attributed to removal of surface sorbed species. Under H_2, with the measurement carried out to higher temperature, the material undergoes complete reduction to yield metallic Mo.

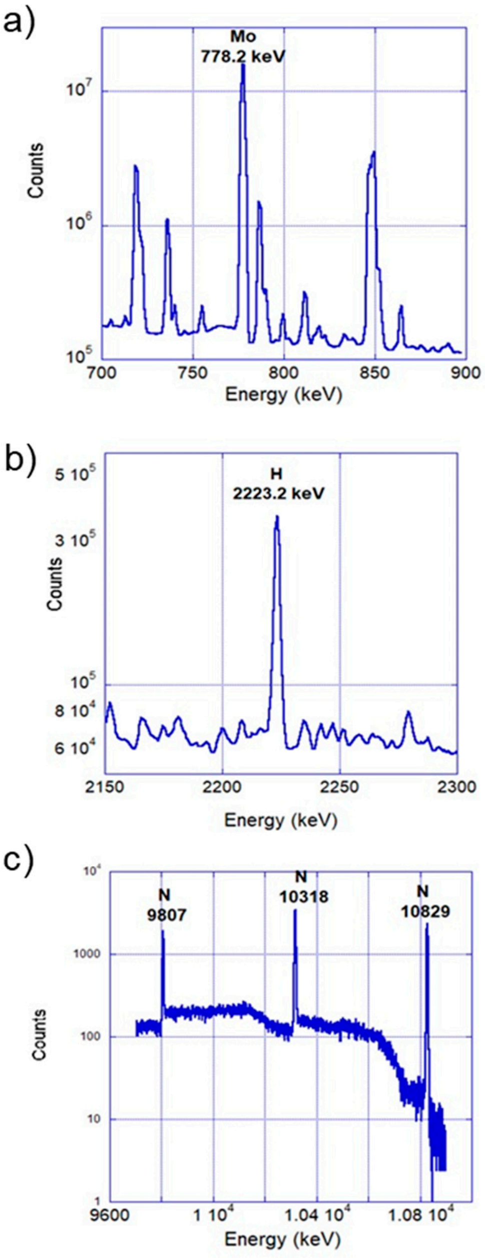

Figure S5: PGAA spectra in the (a) Mo, (b) H, and (c) N regions obtained from δ-$MoO_xN_yH_z$ produced by the ammonolysis of MoO_2 in the presence of steam (main text Table 1, condition 7).

Table S1 *Features of the Mo3d, Mo3p-N1s and O1s peaks detected by thermal XPS (250 ℃) in δ-MoO_xN_y produced by the ammonolysis of MoO_2 in the presence of steam (main text Table 1, condition 7). Mo oxidation states were assigned to the Mo3d peak positions according to the work of Choi and Thompson [32].*

	Binding Energy (eV)	Peak Area (arb. units)
Mo3d$_{5/2}$		
$Mo^{\delta+}$	228.7(1)	129865
Mo^{4+}	230.3(1)	32109
Mo^{6+}	232.2(1)	46835
**Mo3p$_{3/2}$-N1s**		
$Mo^{\delta+}$	394.6(1)	118238
N1s	397.4(1)	98572
Mo^{4+}	395.9(1)	6742
Mo^{6+}	399.8(1)	8912
**O1s**		
O1s	530.3(1)	73366
Surface species	531.9(1)	18298
**Cation/ Anion ratio***	0.77	
**N/O ratio#**	1.34	
**Surface Oxide/Bulk ratio&**	0.61	

* - Area(Mo3p$_{3/2}$$^{(\delta+)+(4+)+(6+)}$) / (Area(N1s)+Area(O1s))

\# - Area(N1s)/Area(O1s)

& - Area(Mo3d$_{5/2}$$^{(4+)+(6+)}$)/Area(Mo3d$_{5/2}$$^{\delta+}$)

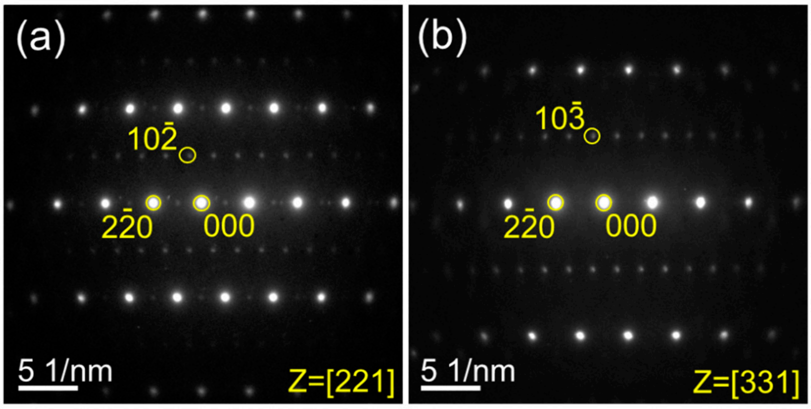

Figure S6: SAED patterns collected along the (a) [221] zone and (b) [331] axes of a crystallite of δ-MoO_xN_y produced by the ammonolysis of MoO_2 in the presence of steam (main text Table 1, condition 7).

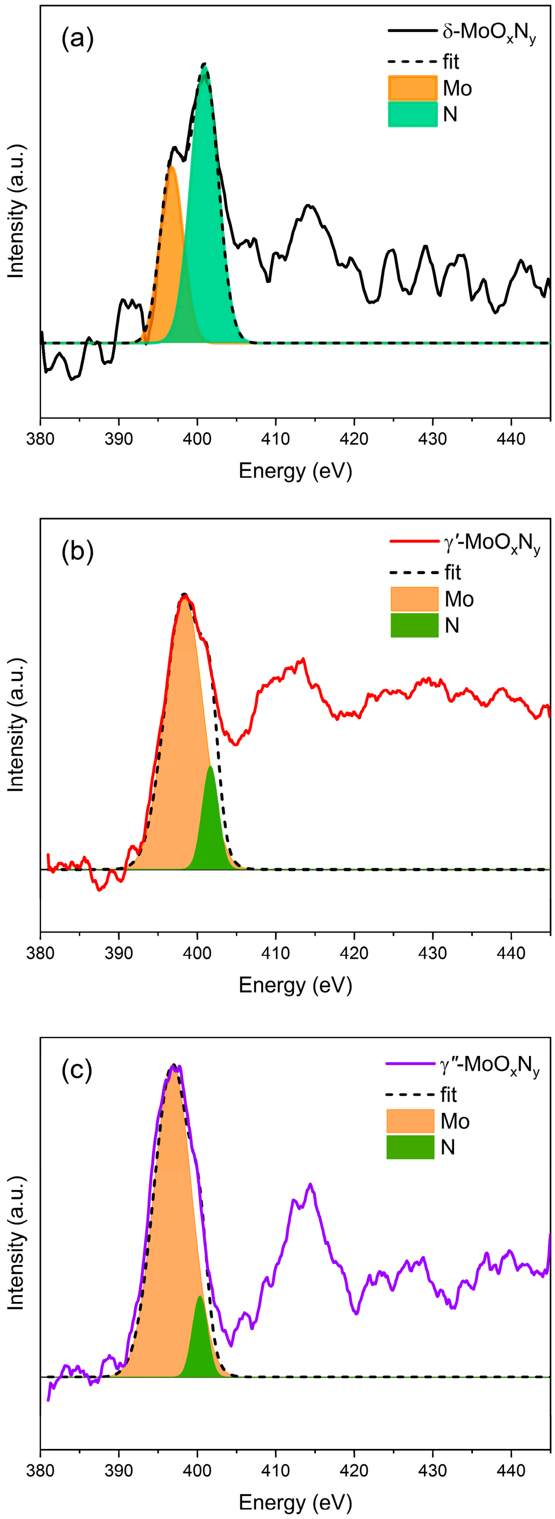

Figure S7: ELNES of Mo-M2,3 edges of molybdenum oxynitrides obtained by ammonolysis: (a) δ-MoO_xN_y (this work, condition 7 of Table 1, main text) (b) γ'-MoO_xN_y, and (c) γ''-MoO_xN_y. Results in (b) and (c) taken from our previous work.[7] Gaussian functions were used to fit the peaks of molybdenum and nitrogen.

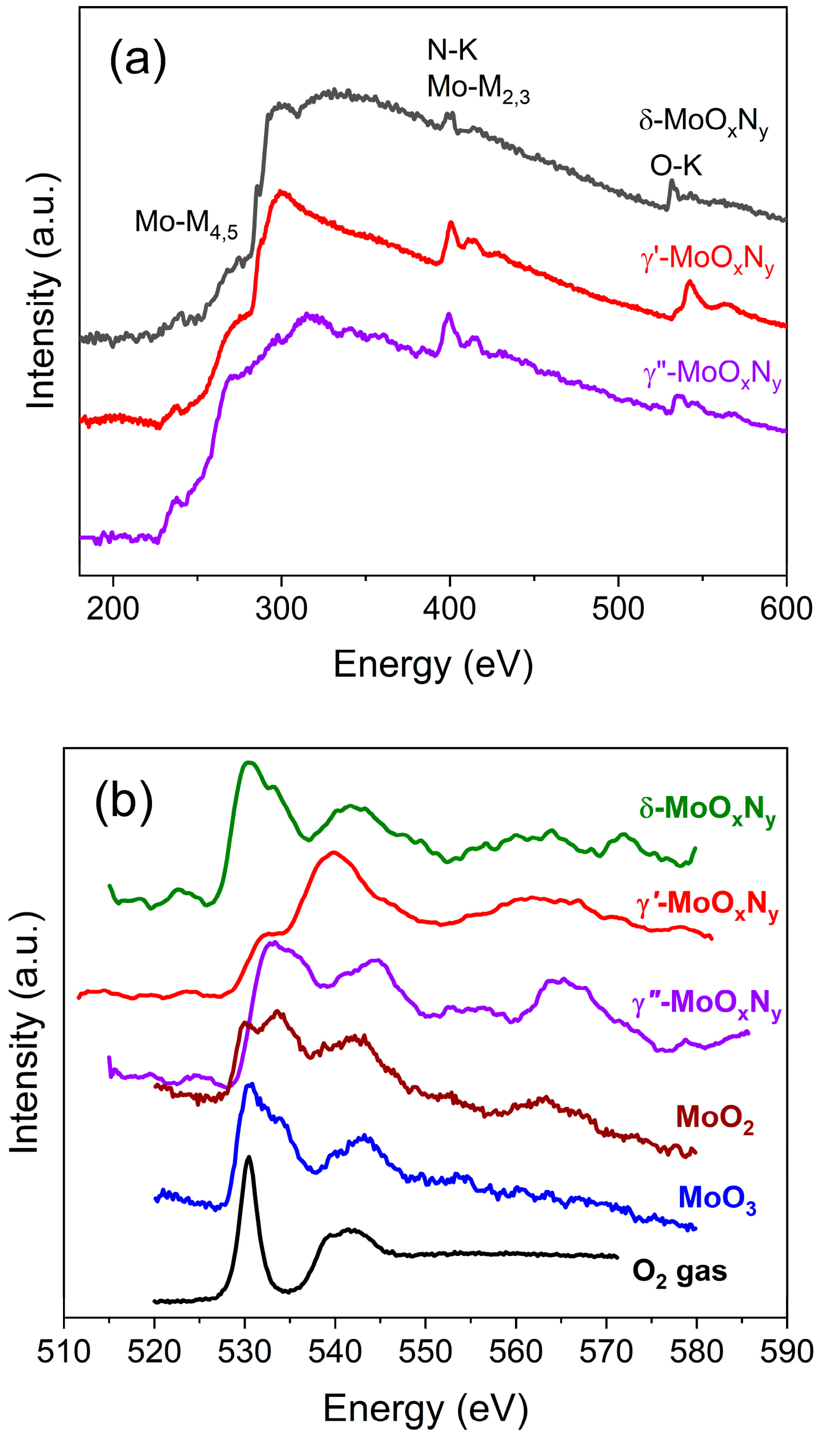

Figure S8: Electron energy loss spectroscopy study of Mo bearing compounds: (a) EELS of δ-MoO_xN_y (this work, condition 7 of Table 1, main text), γ'-MoO_xN_y[7], and γ''-MoO_xN_y[7]; and (b) ELNES of O-K edges of compounds in (a) and of oxygen gas[37,40], MoO_3[41], and MoO_2[41].

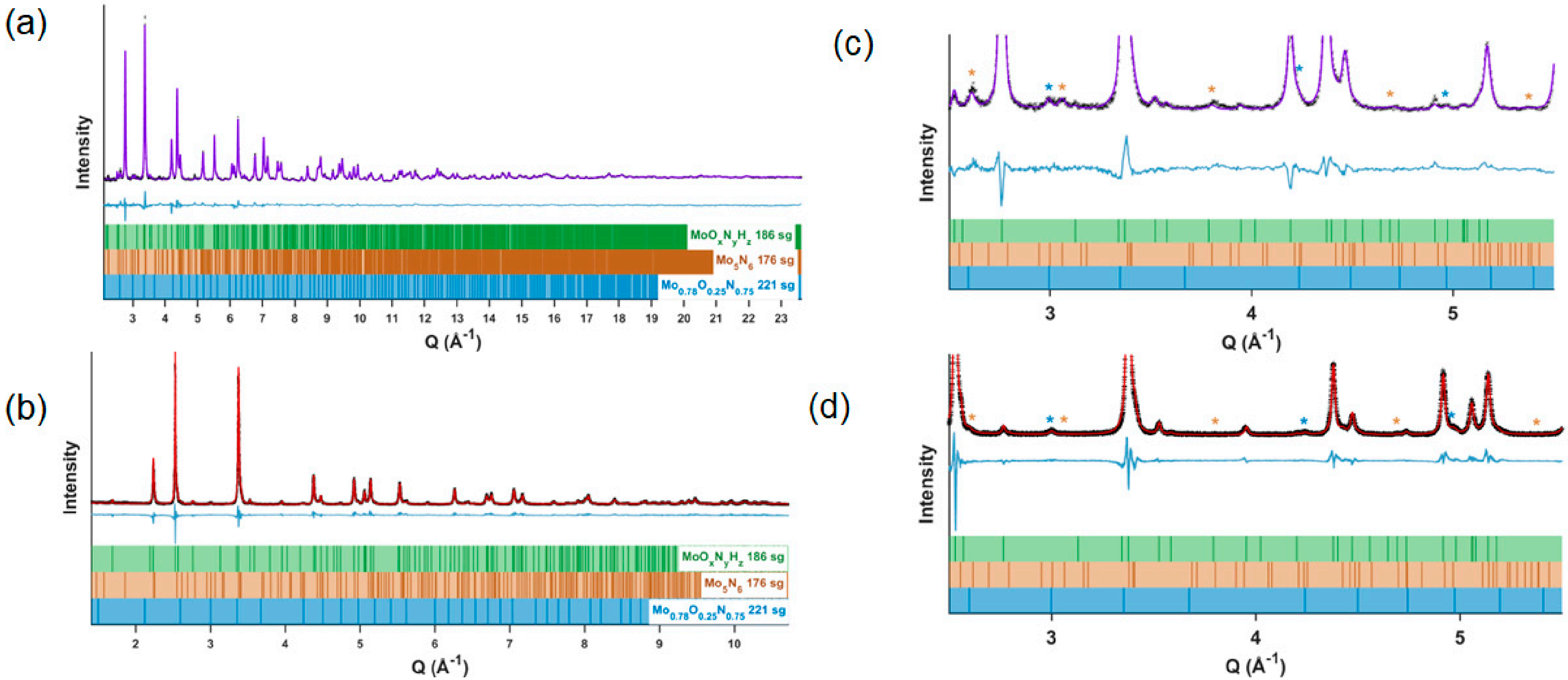

*Figure S9: Measured and calculated powder diffraction patterns, the latter from co-refinement of δ-$MoO_xN_yH_z$ using combined (a,c) neutron powder diffraction and (b,d) X-ray synchrotron data. Measured NPD data in (a) and (c) are shown as black crosses (×) and calculated patterns as solid purple lines. Measured XRD data in (b) and (d) are shown as black plus symbols (+) and calculated patterns as solid red lines. Below each plot, the peak positions for the main phase ($P6_3mc$, 89.19(9) wt.%, green), the secondary Mo_5N_6 phase ($P6_3/m$, 9.07(8) wt. %, brown) and the secondary γ-MoO_xN_y phase ($Pm\bar{3}m$, 1.84(4) wt. %, blue) are shown as vertical lines. Visible secondary phase peaks that do not overlap with those of the main phase are marked with * in (c) and (d). Rietveld refinement residuals: NPD 3.588 %, XRD 14.966 %, overall wR = 7.837 %, and GOF = 1.62. The fit differs from the result in Figure 9 (main text) in that the impurity phase Mo_5N_6 is modeled using a structure with a different space group.*

Table S2. *Summary of the Rietveld co-refinement of neutron time-of-flight powder diffraction and synchrotron X-ray diffraction data collected from δ-$MoO_xN_yH_z$ produced by the ammonolysis of MoO_2 in the presence of steam (Table 1, condition 7). Estimated uncertainty in the final digit(s) of refined values are provided in parentheses. The crystallographic information for the secondary phases was taken from the literature.[7,30]*

Space group	*P*6₃*mc* (186)
Lattice parameters (Å)	*a* = *b* = 5.73834 (13) |*c* = 5.61840 (7)
Composition	$MoO_{0.109(8)}N_{0.891(8)}H_{0.011(5)}$
Density (g/cm³)	9.135

**Site | Element**	**x**	**y**	**z**	**Occupancy**	**Uiso, × 10² Å²**
Site 2(a), 3m | Mo(1)	0	0	0.0 (fixed)	1 (fixed)	0.18(2)
Site 6(c), m | Mo(2)	0.48886(4)	0.51114(4)	0.0014(5)	1 (fixed)	0.042(6)
Site 2(b), 3m | N(1)	1/3	2/3	0.2768(8)	1 (fixed)	0.294(4)
Site 6(c), m | N(2) + O(2) (net occupancy fixed to 1)	0.1670(3)	0.8330(3)	0.7448(8)	0.855(10) N<br>0.145(10) O	0.294(4)
Site 2(b), m | H(1)	1/3	2/3	0.563(14)	0.044(18)	1.00(fixed)

Secondary phase Mo_5N_6 *P*6₃*/m* (176) [30]	9.07(8) wt. %
Lattice parameters (Å)	*a* = *b* = 4.9172(16) Å | *c* = 11.1548(11)
Density (g/cm³)	8.133
Secondary phase $Mo_{0.78}O_{0.25}N_{0.75}$ *Pm*$\bar{3}$*m* (221)[7]	1.84(4) wt. %
Lattice parameters (Å)	*a* = *b* = *c* = 4.1889(7)
Density (g/cm³)	8.031
Overall sample composition	$MoO_{0.098(7)}N_{0.925(7)}H_{0.010(4)}$
Residual wR (%)	
Neutron	3.588 %, 3019 observations
X-ray	14.966 %, 40182 observations
Combined	7.837 %
Goodness of Fit	1.62

[32] Choi, J.G.; Thompson, L.T. XPS study of as-prepared and reduced molybdenum oxides. *Applied Surface Science* **1996**, *93*, 143-149, doi:https://doi.org/10.1016/0169-4332(95)00317-7.

[7] Pandey, S.A.; Zhang, C.; Ibrahim, D.H.; Goldfine, E.A.; Wenderott, J.K.; dos Reis, R.; Paul, R.L.; Spanopoulos, I.; Kanatzidis, M.; Bedzyk, M.J.; et al. Hidden Complexity in the Chemistry of Ammonolysis-Derived "γ-Mo_2N": An Overlooked Oxynitride Hydride. *Chemistry of Materials* **2021**, *33*, 6671-6684, doi:10.1021/acs.chemmater.1c00617.

[40] Sing, M.; Neudert, R.; von Lips, H.; Golden, M.S.; Knupfer, M.; Fink, J.; Claessen, R.; Mücke, J.; Schmitt, H. The electronic structure of metallic $K_{0.3}MoO_3$ and insulating MoO_3 from high-energy spectroscopy. *Physical review. B, Condensed matter and materials physics* **1999**, *60*, 8559-8568.

[37] Egerton, R.F. *Electron Energy-Loss Spectroscopy in the Electron Microscope*, 3rd ed. 2011. ed.; Springer US: New York, NY, 2011.

[41] Lajaunie, L.; Boucher, F.; Dessapt, R.; Moreau, P. Quantitative use of electron energy-loss spectroscopy Mo-M2,3 edges for the study of molybdenum oxides. *Ultramicroscopy* **2015**, *149*, 1-8, doi:10.1016/j.ultramic.2014.11.002.

[30] Ganin, A.Y.; Kienle, L.; Vajenine, G.V. Synthesis and characterisation of hexagonal molybdenum nitrides. *Journal of Solid State Chemistry* **2006**, *179*, 2339-2348, doi:10.1016/j.jssc.2006.05.025.

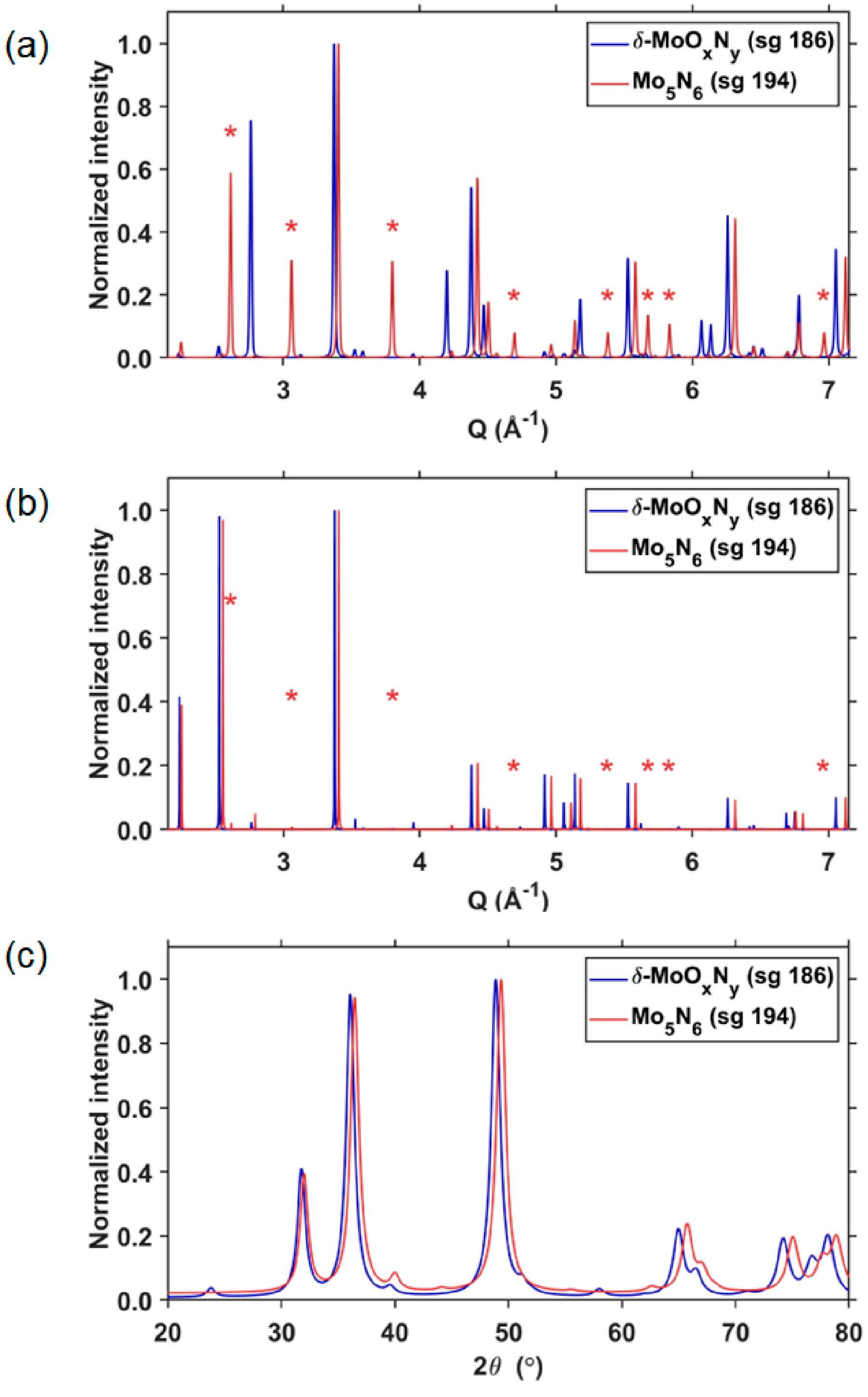

Figure S10: Simulated powder diffraction patterns of δ-MoO_xN_y (space group $P6_3mc$, 186) and Mo_5N_6 phase (space group $P6_3/mmc$, 194) using the radiation sources and instrument parameters for (a) POWGEN neutron TOF, (b) 11-BM X-ray synchrotron, and (c) the laboratory X-ray diffractometer employed in this study. The intensities of the phases have been normalized for all patterns. The most intense peaks of Mo_5N_6 overlap strongly with those of δ-MoO_xN_y. In the NPD patterns, the presence of Mo_5N_6 can be recognized by the presence of peaks indicated with asterisks. In the XRD patterns, the presence of Mo_5N_6 can only be recognized by the presence of shoulders on the main peaks, requiring high resolution measurements. Treatments of Mo_5N_6 in the alternative structure with space group symmetry $P6_3/m$ (176) give essentially identical results.
